# Clinical, cellular, and genomic consequences of a population-enriched *SETD1A* missense variant

**DOI:** 10.21203/rs.3.rs-9900286/v1

**Published:** 2026-07-13

**Authors:** Robert Lease, Rediet T. Oshone, Yumna Ahmed, Samaneh Ali, Sterling Arjona, Jayme Choe, Carlo Colantuoni, Marcia Cortes-Gutierrez, Brian R. Herb, Elizabeth M. Humphries, Evelina Mocci, Rowan O’Hara-Payne, Ryan Kuehner, Coleen Damcott, Hemalatha Sampath, Susan Shaub, Kamah Woelfel, Charlene Wolford, Kwangmi Ahn, Sevilla Detera-Wadleigh, Sander Markx, Joseph A. Gogos, Peter Kochunov, Toni I. Pollin, Teodor Postolache, Alan R. Shuldiner, Francis J. McMahon, L. Elliot Hong, Braxton D. Mitchell, Seth A. Ament

**Affiliations:** 1 Institute for Genome Sciences, University of Maryland School of Medicine, Baltimore, MD; 2 Program in Molecular Medicine, University of Maryland School of Medicine, Baltimore, MD, USA; 3 Program in Cellular and Molecular Biosciences, University of Maryland School of Medicine, Baltimore, MD, USA; 4 Department of Neurology, University of Maryland School of Medicine, Baltimore, MD, USA; 5 Department of Neurology, Johns Hopkins School of Medicine, Baltimore, MD, USA; 6 Department of Pharmacology and Physiology, University of Maryland School of Medicine, Baltimore, MD, USA; 7 Program in Molecular Epidemiology, University of Maryland School of Medicine, Baltimore, MD, USA; 8 Department of Medicine, University of Maryland School of Medicine, Baltimore, MD, USA; 9 Program in Human Genetics, University of Maryland School of Medicine, Baltimore, MD, USA; 10 Lancaster Bible College, Lancaster, PA, USA; 11 Program in Personalized and Genomic Medicine, University of Maryland School of Medicine, Baltimore, MD, USA; 12 Department of Psychiatry and Behavioral Sciences, McGovern Medical School, The University of Texas Health Science Center at Houston, Houston, TX, USA; 13 Human Genetics Branch, Intramural Research Program, National Institute of Mental Health, Bethesda, MD, USA; 14 Department of Psychiatry, Columbia University Irving Medical Center, New York, NY, USA; 15 Mortimer B. Zuckerman Mind Brain and Behavior Institute, Columbia University, New York, NY, USA; 16 Department of Epidemiology & Public Health, University of Maryland School of Medicine, Baltimore, MD, USA; 17 Department of Psychiatry, University of Maryland School of Medicine, Baltimore, MD, USA; 18 Maryland Psychiatric Research Center, Department of Psychiatry, University of Maryland School of Medicine, Baltimore, MD, USA; 19 UM-MIND Institute for Neuroscience Discovery, University of Maryland School of Medicine, Baltimore, MD, USA

## Abstract

Rare variants in *SETD1A*, encoding a histone H3K4 methyltransferase, are among the strongest genetic risk factors for schizophrenia. Exome sequencing (n=3,736) revealed a population-enriched SETD1A missense variant (P596L) in the Lancaster Old Order Amish founder population, presenting a unique opportunity to elucidate variant-specific, multi-scale mechanisms. Psychiatric and cognitive phenotyping revealed nearly two-fold increased risk for bipolar disorder, accompanied by allele dose-dependent cognitive deficits in adulthood. Induced pluripotent stem cells (iPSCs) from homozygous carriers exhibited signatures of SETD1A hypofunction, including reduced proliferation and heightened susceptibility to replication stress and DNA double-strand breaks. During forebrain-directed differentiation, homozygous mutant cells displayed premature activation of neurodevelopmental transcriptional programs but impaired neural rosette formation, reduced neurite complexity, and early progenitor senescence. Multi-omic profiling revealed dysregulation of gene modules converging on replication stress pathways and neuronal regulatory networks enriched for autism and psychiatric risk genes. Pharmacologic inhibition of the H3K4 demethylase KDM5 partially rescued replication stress and neurite deficits, supporting an epigenetic mechanism and suggesting therapeutic tractability. Together, these findings link a population-enriched missense variant to disrupted chromatin regulation, genome stability, and neurodevelopmental timing, bridging human genetic risk with cellular pathophysiology.

## INTRODUCTION

Adult-onset psychiatric disorders such as schizophrenia (SCZ), bipolar disorder (BD), and major depressive disorder (MDD) are strongly heritable^1,2^. Recently, exome sequencing has revealed rare risk variants for these disorders, including ten high-confidence risk genes for schizophrenia^3^ and 102 for autism spectrum disorder^4^. Rare risk variants are valuable for clinical and mechanistic translation, owing to their relatively large effects on disease risk and easier-to-interpret consequences for the functions of protein-coding genes, compared to common risk variants. However, the infrequency of these variants in the general population poses logistical challenges for follow-up studies.

A case in point is *SETD1A*, encoding SET Domain Containing 1A, one of the top SCZ risk genes from large exome sequencing studies^3^. The initial discovery of *SETD1A* as an SCZ risk gene raised several fundamental questions: What clinical phenotypes do SETD1A mutation carriers exhibit, through what mechanisms do these variants influence brain development and function, and how might these phenotypes be targeted for precision therapy? Clinical reports now show that *SETD1A* variants are associated with a syndrome of neurodevelopmental features, including cognitive deficits, developmental delay, and epilepsy, along with increased risk for psychiatric illness, though the presentation is variable and incompletely defined^3,5–7^. In parallel, work in model systems demonstrates that reduced SETD1A levels disrupt cortical development and synaptic function, with mouse and stem-cell studies highlighting altered gene regulation, impaired neuronal maturation, and circuit deficits^8–18^. Yet despite these advances, most mechanistic studies have relied on engineered loss-of-function alleles rather than clinically observed variants, limiting the ability to link specific mutations to cellular phenotypes and clinical outcomes. Moreover, clinical phenotyping has focused largely on children, and systematic characterization of *SETD1A* carriers in adulthood – when psychiatric symptoms emerge – remains scarce. Consequently, major gaps remain in understanding how distinct *SETD1A* variants shape neurodevelopment across the lifespan. Mechanistic follow-up has been even more limited for other recently described SCZ risk genes: Although a few of these genes with well-known functional roles (e.g., *GRIN2A, GRIA3, CACNA1G*) have enabled progress on their rapid investigation, risk genes without a prior neurobiological hypothesis remain almost completely uncharacterized^19–21^.

Multi-scale studies in founder populations represent a unique opportunity to establish variant-level genotype-phenotype relationships^2,22^. In founder populations, genetic drift results in certain otherwise-rare variants becoming much more common than in the broader population. Some of these population-enriched variants have substantial effects on disease risk. Studies of population-enriched variants have proven invaluable in characterizing disease mechanisms and therapeutic opportunities in a variety of common illnesses^23–26^. However, the contributions of population-enriched variants in adult-onset psychiatric disorders are poorly understood^2^.

Here, exome sequencing of 3,736 individuals in the Lancaster Old Order Amish (OOA) founder population revealed an OOA-enriched, deleterious missense variant in *SETD1A*. We performed genotype-first studies to describe its clinical, neurodevelopmental, and genomic consequences.

## RESULTS

### A population-enriched *SETD1A* missense variant associated with cognitive deficits and risk for bipolar disorder

Population isolates provide unique opportunities for clinical deep phenotyping and functional studies of population-enriched variants. We analyzed exome sequences from two Lancaster Old Order Amish (OOA) cohorts to characterize population-enriched protein-coding variants in established risk genes for neuropsychiatric conditions. The Amish Connectome Project (ACP) recruited OOA families multiply-affected with mental health disorders. The Amish Wellness Study (AWS) conducted broad phenotyping of additional individuals from the same OOA parishes, including assessments of mood symptoms, as well as history of clinical diagnoses and medication use. We sequenced the exomes of 89 ACP participants with major depressive disorder (MDD), 22 with bipolar disorder (BD), ten with schizophrenia spectrum disorders (SCZ), 51 with other diagnoses, and 262 with no mental illness. We analyzed these exome sequences together with those of 3,302 unaffected AWS participants who reported no history of mood symptoms, psychiatric diagnoses, or prescriptions for medications commonly used in the treatment of psychiatric disorders.

In these data, we identified 108 OOA-enriched (population-specific MAF > 1%), deleterious (CADD > 10) protein-coding variants in established risk genes for psychiatric or neurodevelopmental disorders. Although our cohort was underpowered for genome-wide association studies, several analyses support impacts on psychiatric risk in the OOA. A generalized linear mixed model controlling for polygenic background in our cohort revealed nominally significant associations with increased risk for BD or MDD for 15 of the 108 variants (*P* < 0.05; **Fig. S1, Tables S1, S2**). This included replication of two previously described OOA-enriched risk variants for mood disorders -- rs78247304 (KCNH7 R394H)^27^ and rs201250006 (CNOT1 M547I)^28^. More broadly, OOA-enriched variants in established neuropsychiatry-related gene sets -- e.g., risk genes for neurodevelopmental disorders, targets of the Fragile X Messenger Ribonucleoprotein -- were more likely to reach nominal significance levels for association with BD or MDD than OOA-enriched variants in other genes (**Tables S3-S5**). Overall, these data suggest that OOA families segregate population-enriched variants in multiple genes previously implicated in risk for psychiatric conditions, potentially valuable for genotype-first callback studies and deep phenotyping.

**Figure 1.**
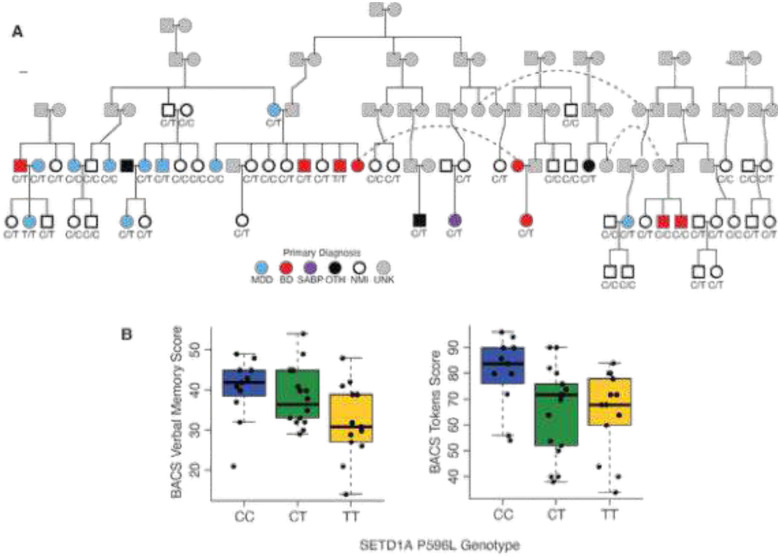
Discovery of a population-enriched SETD1A missense variant associated with cognitive deficits and risk for bipolar disorder. A. The largest connected pedigree in the Amish Connectome Project (ACP) sample segregating SETD1A P596L. BD, bipolar disorder; MDD, major depressive disorder; SABP, schizoaffective disorder, bipolar type; OTH, other diagnoses; UNK, unknown / not assessed; NMI, no mental illness; square, male; circle, female. B. In a genotype-first callback study of SETD1A P596L homozygotes, heterozygotes, and non-carriers, P596L was associated with quantitative, dose-dependent deficits in cognitive domains assessed by the Brief Assessment of Cognition in Schizophrenia (BACS).

We selected rs139187973, henceforth SETD1A P596L, for follow-up for the following three reasons: (i) *SETD1A* is a genome-wide significant risk gene for schizophrenia in the largest exome-sequencing study of SCZ to date (SCHEMA: *P* = 2.0e-12)^3^, and we previously reported a non-OOA pedigree with BD that segregated a distinct *SETD1A* variant^29^. (ii) SETD1A P596L was associated with a 1.8-fold relative risk for BD in our sample (*P* = 0.027; Fig. 1A). (iii) SETD1A P596L was the top variant from multi-parameter prioritization, combining evidence from OOA-specific genetic associations with protein-protein interactions in a gene network centered on established risk genes for neuropsychiatric traits (**Fig. S2**). (iv) SETD1A P596L is exceptionally common in the OOA population, facilitating recruitment of carriers: The variant had an allele frequency of 5.2% in our OOA sample, compared to 0.09% in the broader population (gnomAD exomes; 55-fold enriched), corresponding to 390 copies in our association study of BD, as well as 721 heterozygotes and 28 homozygotes in a broader database of Lancaster OOA who have participated in research studies at our institution.

Little is known about the clinical characteristics of adult carriers of *SETD1A* variants beyond their associations with schizophrenia and related diagnoses. We recruited SETD1A P596L homozygotes (n=15), heterozygotes (n=18), and non-carriers (n=15) from the OOA population to test the hypothesis that carriers present quantitative differences in SCZ- and BD-related cognitive and behavioral dimensions, independent of diagnosis. Carriers performed worse than non-carriers on two subtests of the Brief Assessment of Cognition in Schizophrenia (BACS)^30,31^, including Verbal Memory (*P* = 0.02) and Tokens (*P* = 0.04) subtests (Fig. 1E). We found no differences in performance on other cognitive dimensions assessed by the BACS (**Fig. S3**), nor on symptom domains assessed by the Positive and Negative Syndrome Scale (PANSS)^32,33^; **Fig. S3**). These findings suggest selective disruption of cognitive domains commonly affected in schizophrenia, independent of diagnosis.

Notably, the effects of SETD1A P596L alleles on cognition appeared additive, with homozygotes performing worse than heterozygotes, especially on the Verbal Memory subtest. Our cohort spanned the adult lifespan from 24 to 75 years. As expected, cognitive performance was lower in older participants, and the SETD1A P596L carriers with the lowest scores were >65-years-old (**Fig. S4**). In summary, SETD1A P596L is a population-enriched missense variant associated with bipolar disorder risk and cognitive deficits in specific sub-domains.

### SETD1A P596L results in SETD1A hypofunction in patient-derived induced pluripotent stem cells

We set out to understand the molecular and cellular consequences of SETD1A P596L in human cells. We obtained peripheral blood mononuclear cells from two homozygous carriers and reprogrammed them to produce three clonal induced pluripotent stem cell lines (iPSCs) from each donor. As controls, we studied three iPSC and embryonic stem cell (ESC) lines from non-carriers. Each cell line was validated for the expression of pluripotency markers, pluripotent stem cell morphology, normal karyotype, and SETD1A P596L genotype.

**Figure 2.**
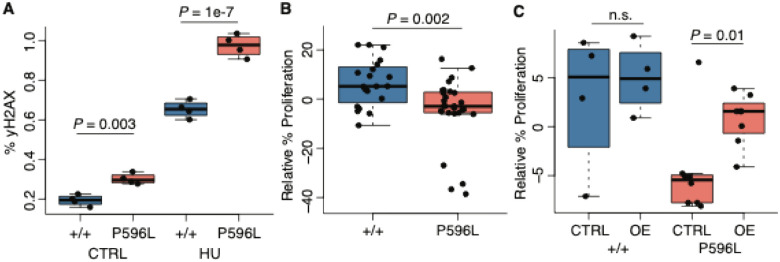
Effects of SETD1A P596L on signatures of SETD1A hypofunction in patient-derived human induced pluripotent stem cells. A. Effects of SETD1A P596L on susceptibility to DNA double strand breaks. Y-axis indicates the proportion of cells positive for the DNA double strand break marker yH2AX, comparing iPSCs from homozygous SETD1A P596L/P596L carriers to non-carriers. CTRL = baseline conditions; HU = cells treated with 4 uM hydroxyurea. B. Effects of SETD1A P596L on cellular proliferation, quantified as the percent (%) of cells incorporating the thymidine analog EdU within a 3-hour period. Y-axis indicates the residual after accounting for batch effects across several groups of cells assayed on different days. C. Cellular proliferation deficits in P596L/P596L iPSCs were rescued by overexpression of reference-sequence SETD1A. CTRL = baseline conditions; OE = *SETD1A* overexpression.

We hypothesized that SETD1A P596L would display cellular signatures of SETD1A hypofunction. Hypofunctional variants of SETD1A have previously been shown to cause increased susceptibility to DNA double strand breaks^6^. To measure this, we assayed the proportion of cells displaying phosphorylation of the Ser-139 residue of the histone variant H2AX (γH2AX), a marker for the induction of DNA double-strand breaks^34^. At baseline, ~30–35% of P596L iPSCs were positive for γH2AX, compared to ~15–20% of +/+ iPSCs (Fig. 2A; ANOVA: *P* = 0.003). As expected, treatment with hydroxyurea induced double-strand breaks in iPSCs of both genotypes. SETD1A P596L genotype was associated with a substantially greater susceptibility to hydroxyurea-induced DNA double-strand breaks, with >90% of P596L iPSCs positive for γH2AX, compared to <70% of +/+ iPSCs (*P* = 1.0e-7).

SETD1A loss-of-function has also been associated with a decreased rate of cellular proliferation^35,36^. We assayed this via incorporation of the thymidine analogue 5-ethynyl-2′-deoxyuridine (EdU). P596L iPSCs proliferated slower than +/+ iPSCs, with a median of ~10% fewer cells replicating over a 3-hour period (ANOVA: *P* = 0.002; Fig. 2B). Consistent with this, P596L iPSCs displayed a slower doubling time, despite their otherwise normal morphology and expression of pluripotency markers.

We performed a rescue experiment to confirm that decreased cellular proliferation arose from SETD1A hypofunction. We transfected P596L and +/+ iPSCs with *in vitro* transcribed reference-sequence SETD1A mRNA at a supraphysiological concentration (250 ng per 12-well dish, delivered via a ribonucleoprotein complex). SETD1A overexpression rescued cellular proliferation to levels comparable to +/+ iPSCs (ANOVA: *P* = 0.01; Fig. 2C). By contrast, SETD1A overexpression had no effect on the proliferation of +/+ iPSCs. Together, these results suggest that SETD1A P596L results in cellular signatures of SETD1A hypofunction.

### Transcriptomic consequences of SETD1A P596L during forebrain neurogenesis

**Figure 3.**
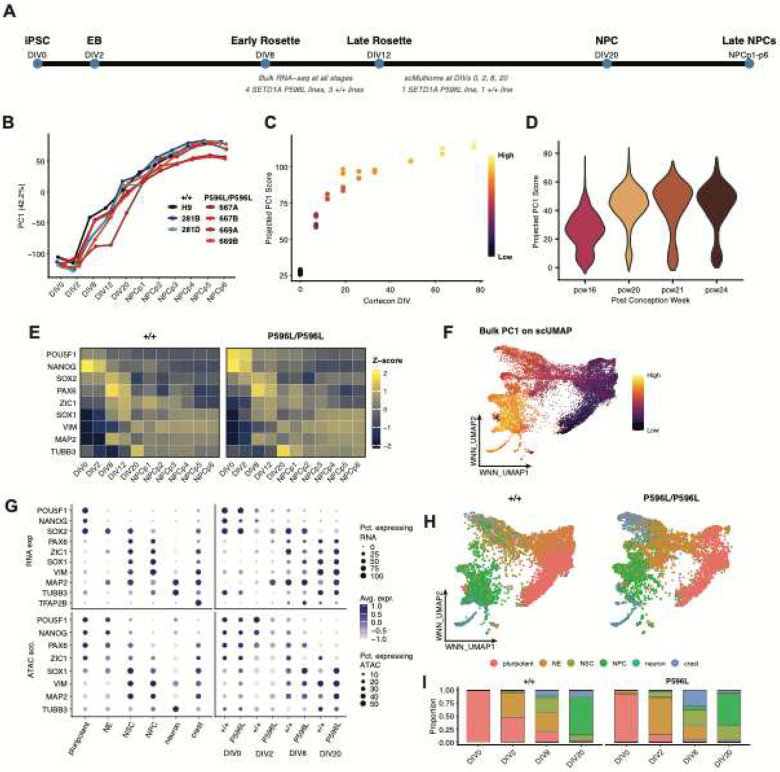
Gene expression signatures of SETD1A P596L/P596L vs. +/+ iPSCs during neural induction. **A.** Neural induction timecourse. **B-D.** Bulk mRNA-seq revealed similar induction of neurogenic genes in all cell lines. The first principal component (PC1) describes a pattern that is upregulated over the course of neural induction, similarly in all cell lines (B). Transfer learning confirmed that PC1 from our data is also up-regulated across neurogenesis in mRNA-seq reference datasets for neural induction of iPSCs in vitro (C) and for prenatal human cortical development (D). **E.** Expression patterns of marker genes for pluripotent cells (*POU5F1, NANOG*), neural stem cells (*SOX2, PAX6, ZIC1*), neural progenitors (*SOX1, VIM*), and neurons (*MAP2, TUBB3*). **F.** Transfer learning confirmed that PC1 is replicated in our scRNA/ATAC Multiome data. **G,H.** scMultiome analysis confirms predicted cell type expression patterns and neurogenic trajectories of P596L/P596L vs. +/+ iPSCs. **I**. Cell type proportions in +/+ vs. P596L/P596L iPSCs at each neural induction timepoint.

The effects of *SETD1A* in the brain are thought to begin during neurodevelopment^13,15,37^. We focused on the early stages of neurogenesis, as complete loss of *SETD1A* in mouse embryos resulted in a failure of neurogenesis at the neural stem cell (NSC) stage^37^. Proliferation of neural stem cells (NSCs) and neural progenitor cells (NPCs) is tightly regulated in the human neocortex and is a important mechanism contributing to the number and types of neurons produced^38^. To model these effects, we differentiated P596L and +/+ iPSC clones to forebrain-specified NPCs via embryoid bodies, using dual SMAD inhibition (Fig. 3A). NPCs were serially passaged to model intrinsic changes that contribute to their neurogenic or gliogenic characteristics^39^.

We first characterized the effects of SETD1A P596L via transcriptomic analyses at multiple stages of neural induction (Fig. 3A), including iPSCs (Day In Vitro [DIV] 0), embryoid bodies (DIV 2), neural rosettes (DIV 8 and 12), and NPCs (DIV 20). In addition, we studied six subsequent time points during serial passaging of the iPSC-derived NPCs (NPCp1-p6). mRNA-seq of pooled cells from each time point was performed for n=4 iPSC lines from homozygous carriers (P596L), compared to n=3 lines from non-carriers (+/+). Principal component analyses revealed that major dimensions of variation were shared among all the lines (Fig. 3B). Transfer learning^40^ from published RNA-seq of neurogenic trajectories *in vitro*^41^ and *in vivo*^42^ indicated that these patterns correspond to the expected induction of neurogenic genes by both P596L and +/+ iPSCs (Fig 3C,D; **Fig. S5**). Marker gene expression confirmed normal responses to neural induction cues through the sequential activation of markers for pluripotency (*POU5F1*, *NANOG*; DIV0), neuroectoderm, (*SOX2*; highest at DIV2), NSCs (*PAX6*, *SOX1*; highest at DIV8 and DIV12), and NPCs (*VIM, TUBB3*; highest at DIV20 and serial passaging timepoints) (Fig 3E).

We further characterized these neurogenic trajectories through single cell RNA/ATAC Multiome sequencing of one line per genotype at DIV 0, 2, 8, and 20 (n=15,126 cells, Fig. 3F). Most cells of each genotype followed the expected neural lineage from pluripotent stem cells (*POU5F1*+) to neuroectoderm (*SOX2*+), NSCs (*PAX6*+/*SOX1*+), NPCs (*VIM+/TUBB3*+), and neurons (*MAP2+*) (Fig. 3G). A smaller proportion of cells were annotated to alternative cell fates such as neural crest (TFAP2B+, arising primarily at day 8). Differential abundance analyses indicated that an increased proportion of SETD1A P596L cells entered the neurral lineage by DIV2 (Fig. 3H,I; **Fig. S6**), while +/+ cells were more likely to still be expressing pluripotency markers (P596L = 14%, +/+ = 47%, p = 6.56e-83). Pseudotemporal trajectory analysis revealed that SETD1A P596L was associated with an accelerated progression through the earliest stages of neural induction, particularly at DIV2. However, non-mutant cells progressed more rapidly at later stages of neural induction and caught up to P596L NPCs by DIV20 **(Fig. S7)**. These bulk and single cell RNA/ATAC Multiome datasets can be explored interactively using the NeMO Analytics web portal^43,44^, alongside reference datasets (https://nemoanalytics.org/p?l=5c559012).

**Figure 4.**
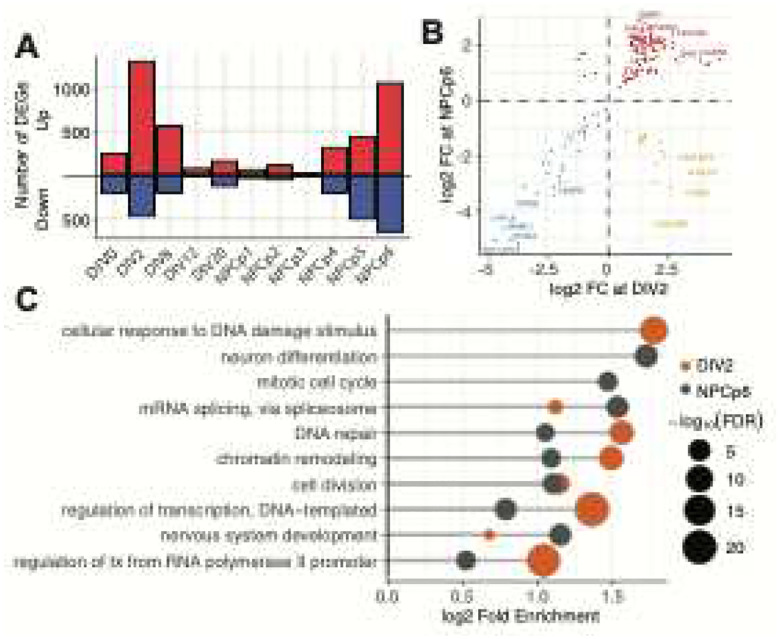
SETD1A P596L is associated with gene expression changes during neurogenesis. **A.** Counts of up- and differentially expressed genes (DEGs; FDR < 0.05, abs(log2FC) > 1) at each neural induction timepoint. **B.** Many DEGs were concordinantly differentially expressed both early in neural induction, during neuroectoderm specification (DIV2) and late in neural induction after multiple passages as neural progenitors (NPCp6). **C.** Across timepoints, DEGs were enriched for Gene Ontology terms related to neurodevelopment, cell cycle regulation and DNA damage responses, and chromatin remodeling.

We analyzed differentially expressed genes (DEGs) at each time point in our mRNA-seq dataset to assess more specific changes during neurogenesis. DEGs were detected primarily at DIV 2 (1,738 DEGs; FDR < 0.05; |logFC| > 1; Fig 4A; **Table S6**) and NPCp6 (1,517 DEGs), corresponding to the earliest and latest stages of differentiation. Fewer DEGs were detected at intermediate timepoints, potentially due to greater line-to-line variation. Comparing the DEGs detected across timepoints revealed both shared and distinct effects of SETD1A P596L at DIV2 vs. NPCp6 (Fig 4B). DEGs were over-represented (FDR < 0.05) for functional categories that suggested effects on the neurodevelopmental process (e.g., “positive regulation of axon extension”), as well as cellular proliferation and DNA damage responses (e.g., “cellular response to DNA damage,” “regulation of DNA repair,” and “cell cycle checkpoint”; Fig 4C; **Table S7**). Among DNA damage signaling pathways, we observed the strongest activation at DIV2 of genes involved in homology directed repair, while pathways activated primarily at later timepoints included genes related to Fanconi Anemia, single strand break repair, and replication fork stress **(Fig. S8)**. Collectively, these results suggest that SETD1A P596L is associated with differences in the timing and efficiency of neurogenesis, including gene expression changes related principally to the regulation of neurodevelopment, as well as the regulation of DNA damage and cellular proliferation.

### SETD1A P596L is associated with accelerated yet inefficient neural induction

**Figure 5.**
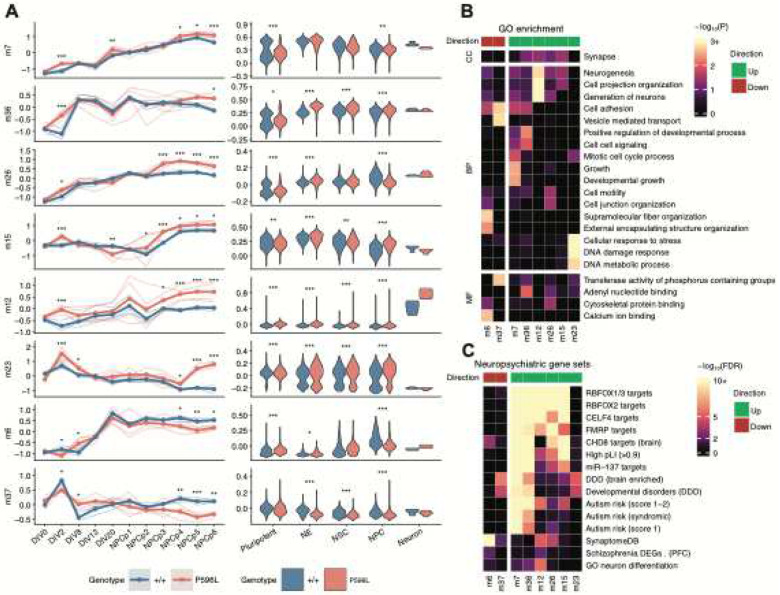
Gene co-expression modules define stage- and lineage-specific programs perturbed by SETD1A P596L across neural induction. **A. Left**: Expression patterns of eight gene modules (entroids ± SEM) in the bulk mRNA-seq time course. **Right**: expression patterns of the same modules across cell types in scMultiome data, stratified by genotype. Asterisks indicate genotype effects within the indicated stage or cell type (*P<0.05, **P<0.01, ***P<0.001). **B.** Enrichment of modules in Gene Ontology (GO) gene sets. **C.** Enrichment of modules in curated neuropsychiatric and neuronal regulatory gene sets.

We applied a network biology approach to integrate our mRNA-seq and scMultiome datasets and extract testable hypotheses for functional experiments. K-means clustering revealed eight gene co-expression modules that were differentially expressed in P596L vs. +/+ iPSC-derived neural cells in our mRNA-seq dataset (FDR < 0.05) and showed comparable expression changes in our scMultiome data (Fig. 5A, **Tables S8, S9**). Two of these eight modules displayed decreased expression in P596L lines, while six displayed elevated expression. All of the modules also displayed changes in expression over the course of neural induction, with varying temporal dynamics.

Functional annotation of the eight modules suggested multiple, distinct effects of SETD1A P596L on cellular processes, which we validated by independent techniques. The two down-regulated modules had different functions. Module m6 was enriched for gene sets involved in cytoskeletal organization, extracellular matrix, and developmental morphogenesis, including components of intermediate filaments, adhesion molecules, and matrix proteins (e.g., *VIM*) that shape the NPC niche. Module m37 was enriched for GO terms related to endo-lysosomal compartments and protein transport, including endosomal membrane proteins, lysosomal enzymes, and lipid transporters, indicating a role for m37 in metabolic and organelle dynamics in neural cells.

Five of the six up-regulated modules -- m7, m36, m12, m15, and m26 – also increased in expression over the course of neural induction, suggesting accelerated activation of transcriptional programs associated with neural induction. Consistent with this, these modules were enriched for multiple neurodevelopment-related Gene Ontology terms (Fig 5B, **Tables S10, S11**). m7 and m36 displayed the strongest effects early in neural induction and were enriched for functional categories related to general aspects of cellular development such as “regulation of cell–cell adhesion” (m7), “regulation of developmental growth” (m7), “positive regulation of developmental process” (m36), and “cell–cell signaling” (m36). m12, m15, and m26 had the largest effect sizes at later stages of neural induction and were enriched for biological processes more specific to neurons and neurodevelopment, including “cell projection organization” (m12), “neurogenesis” (m12), “synapse organization” (m15), “dendrite development” (m15), and “cell junction organization” (m15), and “post-synapse” (m26). These five modules were also enriched for curated gene sets implicated in neurodevelopmental and psychiatric disorders, including many genes mutated in autism and related disorders (Fig. 5C, **Table S12**). The strongest convergence was on targets of neuronal RNA-binding proteins. Modules m7, m12, m15, m26, and m36 overlap extensively with genes regulated post-transcriptionally by RBFOX1/2 and CELF4, splicing and translational factors linked to autism and epilepsy. Thus, SETD1A P596L–disrupted modules preferentially intersect gene networks involved in neurodevelopment and regulated by key transcriptional and post-transcriptional factors. These results also reinforce that many of the gene expression differences in P596L neural cells correspond to accelerated activation of neuronal gene expression signatures.

**Figure 6.**
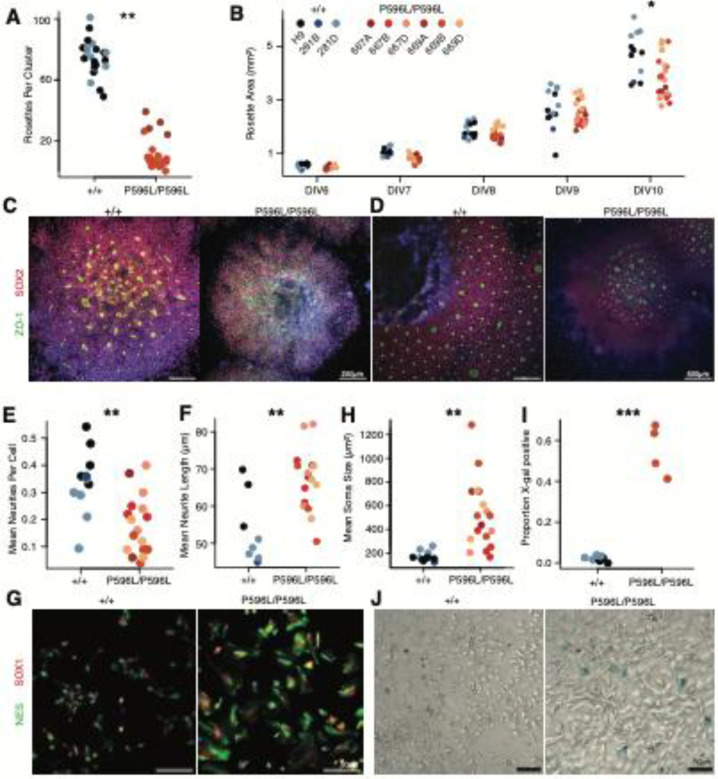
Effects of SETD1A P596L on neural stem and progenitor cell morphology. SETD1A P596L/P596L vs. +/+ iPSCs were differentiated to neural progenitors, via neural rosettes. At the rosette stage (A-D), P596L iPSCs displayed fewer rosettes per cluster (A), as well as reduced rosette area (B). Representative images at DIV8 (C, 10x magnification, scale bar = 100 μm) and DIV 12 (D, 4x magnification, scale bar = 100 μm). At the NPC stage (E-J), SETD1A P596L/P596L was associated with fewer (E), longer (F) neurites per cell. After several passages, SETD1A P596L/P596L NPCs displayed bloated morphology with enlarged soma (H). A high percentage of these SETD1A P596L/P596L NPCs expressed β-galactosidase (X-gal, I), a marker for cellular senescence. Representative images for neurite and soma staining (G, 20x mag, scale bar = 50 μm) and X-gal detection (J, 20x magnification, scale bar = 50 μm)

It is unclear how the accelerated activation of neurodevelopmental gene networks would affect the morphological and functional characteristics of neural cells. To assess morphology, we imaged P596L vs. +/+ NSCs and NPCs. At DIV6–12, all lines were capable of forming neural rosettes, yet P596L was associated with reduced numbers of rosettes per cluster (P = 1.5e-3; Fig. 6A). Moreover, the formation of rosette structures was often delayed, and rosettes expanded more slowly with a significantly smaller area by DIV10 (P = 0.046; Fig. 6B–D). In addition, at the NPC stage (DIV20 and NPCp1-6) we found differences in the morphology of neurites. These included a significant decrease in the average number of neurites per cell (Fig. 6E, P = 1.3e-3), whereas individual neurites were longer (Fig. 6F, P = 8.3e-3).

These findings indicate that premature activation of neurogenic transcriptional programs does not translate into enhanced neural differentiation. Instead, accelerated gene expression is associated with impaired rosette organization and altered neurite architecture, consistent with reduced efficiency or coordination of neural induction. The decreased proportion of NPCs extending neurites further supports the interpretation that early transcriptional acceleration disrupts, rather than improves, the progression of neural development.

### SETD1A P596L is associated with neural progenitor senescence

The remaining up-regulated module, m23, was enriched for gene sets related to “DNA damage response” and “cell cycle checkpoint” and included hub genes such as *BRIP1*, *POLQ*, and *FANCI*. In +/+ lines, this module displayed a gradual decrease over the course of neural induction. By contrast, P596L lines displayed spikes in expression at DIV2, as well as in serially passaged NPCs (NPCp4-p6). Our scMultiome data indicated that m23 expression was also variable across the cell cycle, showing elevated activation during S phase (**Fig. S9**). P596L lines displayed increased expression of m23 even after adjusting for cell cycle phase. As noted above, susceptibility to DNA double strand breaks is a hallmark of SETD1A hypofunction, and these results suggest a spike in sensitivity at these time points.

These observations were supported by changes in the expression of key pathways associated with DNA double strand break repair. At DIV2, P596L cells exhibited significant upregulation of genes such as *RAD51*, *BRCA2*, and *ATR*, which are integral to activation of homologous recombination and signal transduction in response to DNA double-strand breaks **(Fig. S7)**. P596L cells also showed activation of genes involved in nucleotide excision repair pathways, indicative of a broader response to persistent DNA lesions. Genes involved in the cellular stress response became reactivated at later NPC passages, consistent with an increased susceptibility to replication stress (**Fig S7**).

To assess the cellular correlates of these gene expression signatures, we examined the morphology of serially passaged NPCs, focusing on cellular signatures of DNA replication stress. Beginning at NPCp3, many P596L NPCs began to take on a flat, bloated appearance (Fig. 6G), with enlarged soma (Fig. 6H, P = 8.4e-3). These morphological traits are hallmarks of cellular senescence, a stable and normally irreversible state of growth arrest that occurs in response to various forms of cellular stress or damage, preventing further proliferation but maintaining metabolic activity and secretory functions^45^. To confirm senescence, we performed immunohistochemistry in a subset of replicates to quantify GLB1, encoding beta-galactosidase (X-gal), a lysosomal enzyme enriched in senescent cells. ~50% of P596L NPCs at passage 3 expressed beta-galactosidase, compared to fewer than 5% of +/+ NPCs (Fig. 6I,J, P = 1.1e-5). Comparing soma size vs. X-gal staining in P596L NPCs (n=229 cells) revealed that these measures of senescent morphology were strongly correlated (**Fig. S10A**): Soma size predicted X-gal+ staining with an area under the receiver operator curve (AUROC) of 0.87, and at a conservative threshold, 95% of NPCs with a soma size larger than 685 uM^2^ were X-gal+ (**Fig. S10B**). Applying this threshold to the full soma size dataset suggested that at least ~20–60% of NPCs from each P596L line become senescent after 3–6 serial passages, whereas this was never observed in +/+ NPCs (**Fig. S10C**). These results suggest that serial passaging of P596L NPCs causes premature onset of cellular senescence.

### SETD1A P596L dysregulates gene networks overlapping targets of neurodevelopmental and DNA damage-related transcription factors

**Figure 7.**
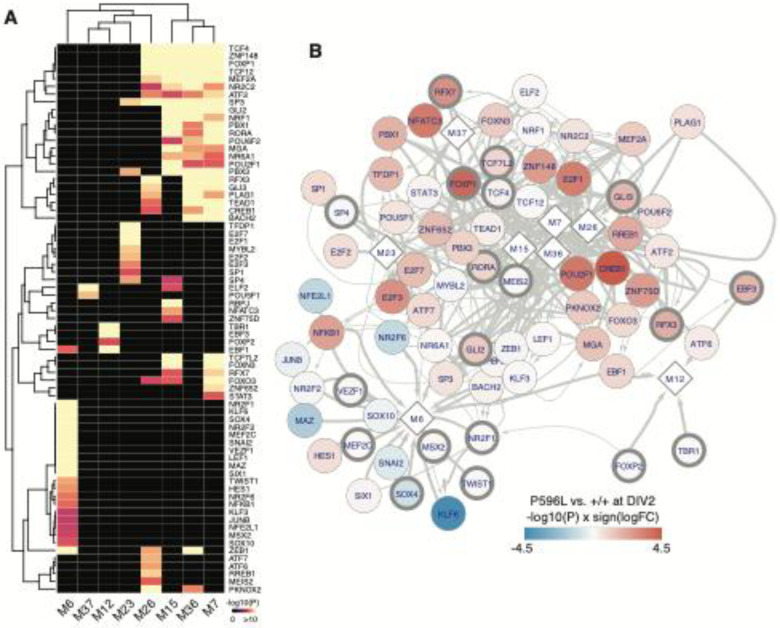
Master regulator transcription factors within SETD1A-associated gene networks. Gene regulatory network (GRN) modeling was performed to predict regulons (target genes) for 383 transcription factors in the context of neural induction. A. Hypergeometric overlap of regulons with eight gene co-expression modules dysregulated by SETD1A P596L. B. TF-to-TF and TF-to-module regulatory interactions predicted by the GRN model. Node color indicates differential expression of each TF at DIV 2. TFs that are established risk genes for neurodevelopmental disorders are indicated by thick borders.

*SETD1A*’s enzymatic function in chromatin regulation suggests gene regulatory changes as direct mechanisms through which SETD1A P596L alters neurodevelopment. To explore this, we defined the upstream regulatory architecture of P596L-associated transcriptional changes by reconstructing a gene regulatory network (GRN), integrating the RNA and ATAC data from our scMultiome dataset. Accessible chromatin regions proximal to each gene, as well as co-accessible distal regions, were used to predict transcription factor occupancy, and regulatory relationships were inferred using a random forest regression model implemented with GENIE3^46–48^. This analysis yielded a GRN model encompassing 383 transcription factors and 10,551 predicted target genes (**Table S13**). We next summarized regulatory interactions at the level of gene co-expression modules to identify candidate master regulators (Fig. 7A; **Table S14**). Each module was associated with a median of 18 significant transcriptional regulators, the majority of which were predicted to act in an activating manner based on positive expression correlations.

We applied this systems-level reconstruction to characterize key transcription factors within SETD1A-associated modules. Predicted master regulators of the cell-cycle and DNA damage module m23 aligned with established regulators of proliferation and genome stability. These included multiple E2F family members and MYBL2, consistent with their known roles in S-phase progression and G2/M control^49^. By contrast, the predicted regulators of neurodevelopmental modules -- m7, m36, m12, m15, and m26 -- overlapped established regulators of neuronal lineage specification and maturation, including TCF4, FOXP1, RORA, and MEF2A^50–53^. These results suggest that cell-cycle and neurodevelopmental modules are controlled by largely distinct sets of canonical transcription factors, corresponding to separable regulatory programs for proliferation/DNA damage responses and neurogenesis. Notably, 19 of the master regulator TFs within P596L-associated gene networks are themselves risk genes mutated in neurodevelopmental disorders, demonstrating convergence on disease-associated gene networks involved in both of these processes (Fig. 7B).

A small number of transcription factors bridged the otherwise distinct transcriptional programs related to proliferation and neurogenesis. For instance, E2F7 was predicted to activate core checkpoint and replication genes within m23, including *CLSPN*, *CHEK1*, *POLE*, and FANC family members. In parallel, E2F7 was predicted to activate neuronal differentiation genes within module m15, including *SUZ12*, *KIF13B*, and *ORC2*. This result was validated by independent, ChIP-seq-derived E2F7 target genes in K562 cells, which strongly overlapped our computationally-predicted E2F7 target genes (OR = 1.9, *P* = 1.2e-6), as well as enrichments in both m23 (OR = 10.7, *P* = 4.5e-37) and m15 (OR = 1.8, *P* = 0.001). Similarly, SP3, SP4, ZNF148, and PBX3 targeted canonical cell-cycle genes in m23 (*FBXO5*, *CDC45*, *SKA1*) alongside neurodevelopmental genes involved in neuronal maturation and morphogenesis (*DLG1*, *RBFOX2*, *CHD2*, *OPHN1*), supporting their roles as shared regulatory nodes spanning proliferative and neuronal programs (Fig. 7B). Together, these findings support a layered regulatory organization in which SETD1A P596L perturbs both module-specific transcriptional programs and a smaller set of shared transcription factors that connect cell-cycle regulation, DNA damage responses, and neurodevelopmental gene expression.

### Epigenetic rescue of SETD1A P596L-associated neurodevelopmental phenotypes

**Figure 8.**
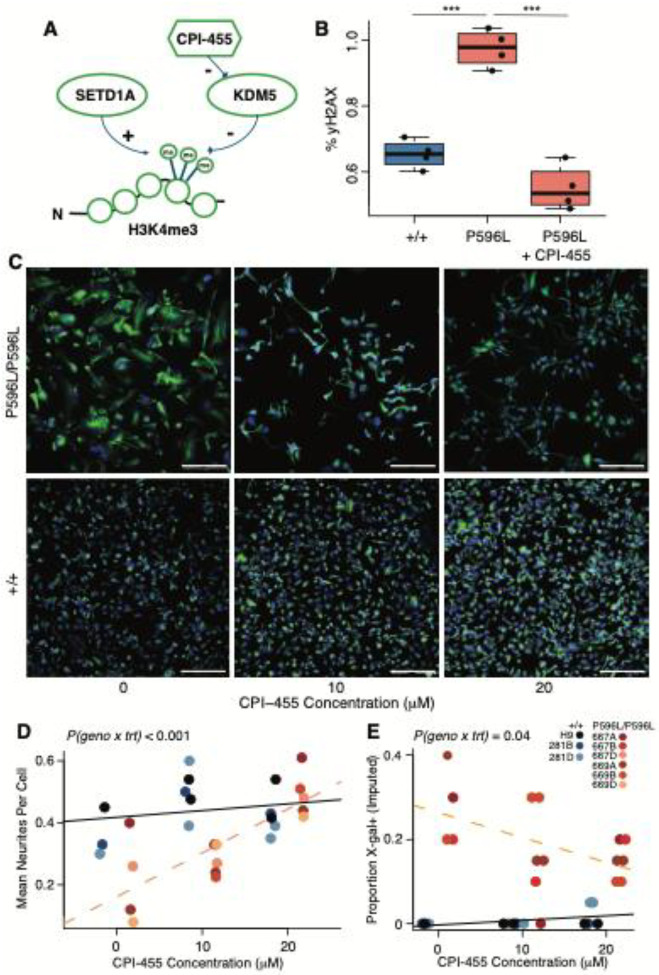
KDM5 inhibition rescues a subset of neurodevelopmental abnormalities associated with SETD1A hypofunction. A. Pharmacological approach to restore H3K4 methylation in the presence of SETD1A hypofunction: inhibition of the H3K4me3-specific histone lysine demethylase KDM5 with a small molecule, CPI-455. Trend lines indicate linear regression on +/+ (blue) vs. P596L/P586L (orange) NPCs. B. CPI-455 treatment (20 uM) rescues DNA double strand break susceptibility in SETD1A P596L/P596L vs. +/+ iPSCs. All groups were treated with 4uM hydroxyurea. Note: Data from cells that were not treated with CPI-455 (left and middle boxes) are replotted from Fig. 2. C–F. Effects of CPI-455 on the morphology of SETD1A P596L/P596L vs. +/+ NPCs at DIV 30. C. Representative images of NPC morphology (20x magnification, scale bar = 50 μm). D. Effect of CPI-455 on the number of neurites per NPC. E. Effect of CPI-455 on the proportion of NPCs with senescent cell morphology, assessed by a soma size larger than the threshold at which we observed 95% of cells to express the senescence marker X-gal (**Fig. S10**).

We hypothesized that the cellular effects of SETD1A^P596L^ were mediated by deficits in H3K4me3, resulting from a decrease in SETD1A’s enzymatic function to write this chromatin modification. To test this, we treated cells with CPI-455, a small molecule inhibitor of the H3K4-specific lysine demethylase KDM5^54^, an approach designed to restore global H3K4 methylation levels reduced due to *SETD1A* hypofunction^9,15^ (Fig. 8A). First, we evaluated our strategy on a known H3K4me3-dependent phenotype: susceptibility to DNA double strand breaks^55^. In iPSCs, we confirmed that 20uM CPI-455 rescued hydroxyurea-induced DNA double-strand breaks (marked by yH2AX) (Fig. 8B).

We inhibited KDM5 continuously throughout neural induction to test whether this rescued cellular neurodevelopmental abnormalities associated with SETD1A P596L. The morphology of P596L NPCs treated with CPI-455 appeared much more similar to that of +/+ NPCs (Fig. 8C, top). By contrast, CPI-455 had no visually apparent effects on the morphology of +/+ NPCs (Fig. 8C, bottom). Quantification of these images confirmed a CPI-455 concentration-dependent rescue in the average number of neurites per cell (Fig. 8D, P(genotype x treatment) < 0.001). In addition, CPI-455 treatment resulted in a dose-dependent decrease in the number of P596L NPCs with senescent cell morphology, assessed via soma size (Figs. 8E. **S10**; P(genotype x treatment) = 0.04). However, 10–20% of NPCs developed senescent morphology despite CPI-455 treatment, indicating only a partial rescue of this phenotype at the tested doses. Also, the effects of KDM5 inhibition on neurite length and rosette morphology were variable across replicates and did not reach statistical significance. These results indicate that some but not all SETD1A P596L cellular phenotypes can be rescued through inhibition of KDM5, most likely via restoration of H3K4 methylation.

## DISCUSSION

In this study, we identified and functionally characterized a population-enriched *SETD1A* missense variant, P596L, associated with bipolar disorder risk and quantitative cognitive deficits in the Old Order Amish founder population. By integrating human genetics, stem cell modeling, multi-omic profiling, and network reconstruction, we demonstrate that SETD1A P596L produces cellular and transcriptional phenotypes consistent with partial loss of SETD1A function and implicate epigenetic dysregulation as a central mechanism linking risk variation to altered neurodevelopmental trajectories.

*SETD1A* is among the most robust rare variant risk genes for schizophrenia, yet the clinical spectrum associated with non-truncating variants remains incompletely understood. The enrichment of P596L in the Amish population enabled quantitative phenotyping across genotype groups independent of categorical diagnosis. Carriers showed additive impairments in verbal memory and executive function without broad symptom differences, suggesting that *SETD1A* variation contributes to dimensional cognitive vulnerability across psychiatric disorders. These findings extend prior reports of *SETD1A* loss-of-function variants^3,5,6^ by suggesting that hypomorphic missense alleles can produce measurable cognitive effects in adulthood, even in the absence of psychosis.

Our data suggest selective effects of P596L on cognitive domains commonly affected in schizophrenia and in experimental models of SETD1A loss of function. The Verbal Memory deficit is consistent with impairments in learning and memory processes linked to hippocampal–prefrontal circuits, while reduced Token Motor performance may reflect slowed psychomotor and processing speed associated with fronto-striatal network function^56^. Notably, these results extend prior studies of SETD1A haploinsufficiency: individuals with pathogenic variants often show developmental cognitive impairment and learning difficulties, and mouse models exhibit deficits in working memory and learning tasks along with abnormalities in prefrontal circuit function^57^. The relative specificity of the observed BACS deficits therefore aligns with convergent evidence that SETD1A disruption preferentially affects neural systems supporting memory formation, cognitive control, and information-processing speed rather than producing a uniform global cognitive impairment.

In patient-derived iPSCs, P596L recapitulated key signatures of SETD1A hypofunction, including increased susceptibility to replication stress and reduced proliferation, which was rescued by SETD1A overexpression. These findings are consistent with prior work implicating SETD1A-mediated H3K4 methylation in genome stability and DNA damage responses^14,35,36,58,59^. The convergence between genetic association and cellular phenotypes strengthens the interpretation that P596L impairs SETD1A enzymatic activity and is not a benign population variant.

Multi-omic profiling revealed that SETD1A P596L alters the temporal dynamics of neural induction. Early stages were characterized by accelerated activation of neurogenic transcriptional modules, whereas later stages exhibited partial convergence with controls. Importantly, premature upregulation of neurodevelopmental gene networks did not enhance differentiation efficiency. Instead, P596L cells displayed impaired rosette organization, altered neurite architecture, and reduced neurite initiation. These findings suggest that early transcriptional acceleration may disrupt coordination of developmental programs, leading to less efficient neural lineage progression. This dissociation between transcriptional activation and morphologic maturation suggests that SETD1A regulates aspects of the timing and integration of neurodevelopmental programs. Such temporal dysregulation may be particularly relevant to psychiatric risk, where subtle alterations in cortical development can have long-lasting circuit-level consequences.

Notably, bidirectional effects on developmental tempo have emerged as recurring phenotypes across iPSC models of neuropsychiatric risk genes, including multiple chromatin regulators^60–64^. In a pooled CRISPRi screen that quantified neuronal differentiation state by pseudotime, repression of several ASD-associated chromatin genes (including *CHD2*, *ASH1L*, and *ARID1B*) shifted cells toward delayed maturation, whereas perturbation of other high-confidence risk genes (including *CHD8*) shifted cells toward accelerated differentiation by the same metric^64^. Complementing these pooled perturbation data, CHD8 loss-of-function models revealed defects in neuroectodermal differentiation and disorganization during early neural lineage progression^63^. Thus, timing effects can manifest as either premature or inefficient specification depending on the gene, allele, and differentiation context. A broader interpretation is that chromatin regulators help establish the “pace” of human neuronal maturation by shaping the accessibility and readiness of transcriptional maturation programs. Overall, these comparisons place SETD1A P596L within an emerging class of risk-associated perturbations that alter developmental timing, while underscoring that accelerated activation of neurogenic gene expression programs can coexist with impaired morphogenesis; i.e., “faster” transcriptional progression is not necessarily equivalent to more effective or better coordinated neuronal differentiation.

Our results also indicate that SETD1A P596L disrupts DNA damage responses, cell-cycle regulation, and proliferative capacity across developmental stages. Mutant cells showed elevated basal γH2AX, hypersensitivity to hydroxyurea-induced replication stress, recurrent activation of replication and checkpoint genes, and reduced proliferation. With serial passaging, neural progenitors developed morphological and molecular hallmarks of cellular senescence. Together, these findings support a model in which SETD1A hypofunction produces chronic replication stress that culminates in premature progenitor arrest.

These phenotypes are consistent with prior mechanistic work establishing SETD1A as a key regulator of genome stability^14,35,36,58^. SETD1A, the catalytic subunit of the COMPASS complex, deposits H3K4me3 at active promoters, but also plays a critical role at replication forks. In yeast and mammalian systems, loss of Set1/SETD1A or H3K4 methylation sensitizes cells to replication stress and impairs fork protection. SETD1A-dependent H3K4 methylation promotes recruitment of repair factors such as FANCD2 and stabilizes stalled forks during S phase^58^. Our observation of elevated DNA breaks and activation of homologous recombination and checkpoint pathways in P596L cells is therefore consistent with a primary defect in replication fork protection. Rapidly dividing neural progenitors may be especially vulnerable to such defects, given the high replicative demand during early corticogenesis. The premature senescence observed in SETD1A P596L NPCs provides a plausible developmental consequence of sustained replication stress. Persistent DNA damage signaling can drive stress-induced senescence, leading to stable cell-cycle arrest. If progenitor pools undergo early senescence, neurogenesis may become quantitatively constrained and temporally dysregulated, potentially establishing a mechanistic connection between genome instability and inefficient neural differentiation.

These findings also align with a broader literature implicating DNA damage and replication-associated mechanisms in neuropsychiatric disorders. Many genes in our DNA stress module are central to DNA repair and checkpoint pathways, and mutations in these genes are known to cause monogenic neurodevelopmental disorders^65–67^. Increasing evidence suggests that replication stress in neural progenitors can promote somatic mutation accumulation, raising the possibility that inherited chromatin variants may create vulnerability to developmental “second-hit” mosaic events^68^. Thus, SETD1A hypofunction may contribute to psychiatric risk not only through transcriptional dysregulation but also by increasing genomic instability during critical windows of brain development. Consistent with this theme, several top schizophrenia risk genes identified by SCHEMA – including *CUL1*, *RB1CC1*, and *HERC1* – encode critical regulators of cell cycle progression and DNA damage response signaling. Collectively, our data reinforce an expanded view of *SETD1A* as a multifunctional chromatin regulator with roles not only in promoter H3K4me3 deposition but also in replication fork protection and coordination of checkpoint signaling. A hypomorphic missense variant such as P596L can therefore plausibly produce convergent effects on genome integrity, cell-cycle control, and neural lineage progression, linking chromatin regulation to replication stress and altered neurodevelopment.

Gene regulatory network reconstruction revealed interactions of SETD1A P596L with two largely separable transcriptional networks: one governing proliferation and DNA damage responses (enriched for targets of *E2F* and *MYBL2* family regulators), and another governing neuronal lineage specification (enriched for targets of *TCF4*, *FOXP1*, *RORA*, and *MEF2A*). The presence of bridging transcription factors -- including *E2F7*, *SP3*, and the schizophrenia risk gene *SP4*^*3*^ -- suggests layered regulatory integration between proliferative and neurogenic programs. This organization supports a mechanism in which SETD1A perturbation does not globally disrupt transcription but instead shifts the balance between canonical cell-cycle and neurodevelopmental regulatory modules. The convergence of master regulators that are themselves implicated in neurodevelopmental disorders further emphasizes shared pathogenic pathways.

Pharmacologic inhibition of the H3K4 demethylase KDM5 partially rescued DNA damage susceptibility and neurite deficits in P596L cells. Although not all phenotypes were fully restored, these findings support a causal role for H3K4 methylation imbalance in mediating at least a subset of SETD1A-associated cellular abnormalities. This mechanism is reinforced by recent work from Erwin and colleagues, who reported that acute effects of SETD1A haploinsufficiency in engineered human neural models can be rescued by KDM5 inhibition^15^. Prior work in *Setd1a*^+/−^ mice demonstrated a complementary therapeutic strategy via inhibition of a distinct histone lysine demethylase, LSD1 (KDM1A)^9^. Collectively, these findings suggest that restoring H3K4me2/3 tone can buffer downstream consequences of SETD1A hypofunction.

These preclinical results have immediate translational relevance because brain-penetrant LSD1 inhibitors are already in clinical development for neuropsychiatric indications, including a Phase IIb program evaluating vafidemstat (ORY-2001) in negative symptoms of schizophrenia^69,70^. While it remains unknown whether LSD1 inhibition will benefit patients specifically carrying mutations in *SETD1A* or other chromatin remodeling genes, the convergence of genetic evidence implicating H3K4 methylation pathways in psychiatric risk and reversibility of core cellular/circuit phenotypes in model systems motivates biomarker-driven clinical translation. Since *SETD1A* mutations are rare, the greatest benefit will emerge if a broader subgroup could be defined. In practical terms, this may entail the development of pharmacodynamic readouts of H3K4 methylation state and DNA damage stress signatures in patient-derived cells, which could enable stratifying future trials by molecular features that reflect SETD1A/H3K4 axis dysfunction rather than diagnosis alone.

At the same time, the partial rescue we observe with KDM5 inhibition suggests important limitations: Variants in *SETD1A* and other chromatin remodeling genes likely perturb multiple layers of regulation -- promoters, enhancers, replication-associated chromatin, and stress responses -- so demethylase targeting may correct only those phenotypes most proximal to H3K4me imbalance. This sets up a testable model for future work: rescue should be strongest for replication stress/DNA damage and select aspects of neurite initiation/architecture, whereas phenotypes driven by longer-range developmental mistiming, accumulated cellular stress, or non-enzymatic SETD1A functions may require combinatorial or earlier intervention.

Several limitations should be considered when interpreting our findings. Although we analyzed multiple independent iPSC clones from carriers and non-carriers, the high GC content of the genomic sequences surrounding the P596L variant precluded generation of a clean isogenic correction line, leaving open the possibility that linked background variation contributes modestly to the observed phenotypes. While the population enrichment of P596L enabled recruitment of one of the largest cohorts of *SETD1A* variant carriers studied to date, the clinical and cellular effects of this specific allele may not generalize to other *SETD1A* missense variants or to truncating mutations associated with schizophrenia. Our *in vitro* neural induction model captures early forebrain development but does not recapitulate later stages of cortical circuit assembly, non-neuronal cell types such as microglia or oligodendrocytes, or the influence of *in vivo* environmental factors. Pharmacologic rescue experiments with KDM5 inhibition demonstrate partial reversibility of selected phenotypes but do not establish long-term functional recovery or *in vivo* efficacy. Together, these considerations highlight the need for complementary approaches – especially the establishment of broader allelic series -- to fully define the pathogenic mechanisms of *SETD1A* variation.

Finally, our study underscores the value of founder populations in identifying and characterizing rare, high-impact variants for psychiatric disorders. In the Lancaster Old Order Amish, the SETD1A P596L allele is markedly enriched (~5% in our sample vs. ~0.09% in the broader population), enabling detection of its association with bipolar disorder and cognitive differences despite a modest cohort size. This enrichment also permitted recruitment of multiple adult homozygous carriers -- an exceedingly rare genotype in the general population -- for detailed cellular analyses. Founder populations thus function as natural genetic experiments, where demographic bottlenecks amplify otherwise rare alleles and facilitate genotype-first phenotyping. Leveraging this context allowed us to move from variant discovery to mechanistic dissection within a single study, a trajectory that is often difficult to achieve in psychiatric genetics.

Importantly, P596L represents only one of a broader set of variants enriched in the Amish population associated with neuropsychiatric traits^22,27,28,71^, including variants in dozens of other well-established neuropsychiatric risk genes. This constellation of population-enriched alleles provides an immediate opportunity to expand mechanistic studies within the same well-characterized cohort. Because multiple carriers can be identified for each variant, it becomes feasible to scale iPSC-based modeling approaches beyond single-gene case studies toward population-level functional genomics. Our groups have already begun systematic derivation of iPSC lines across variant carriers to enable comparative analyses of convergent and divergent cellular phenotypes, integration with deep clinical data, and testing of shared therapeutic strategies^72^. As sequencing efforts expand in genetic isolates, similar frameworks could support scalable, genotype-first platforms that bridge human genetics and experimental neurobiology, accelerating the translation of rare variant discoveries into biological insight and precision approaches in mental health disorders.

## METHODS

### Clinical cohorts.

The Old Order Amish (OOA) population of Lancaster County, PA immigrated from Central Europe in the 1700s^73^. Nearly all of the ~40,000 individuals in the present-day OOA population can trace their ancestry back 12–15 generations to fewer than 750 founders^74^. Investigators at the Amish Research Program at the University of Maryland School of Medicine have been conducting genetics studies in this population since 1993, enrolling a total of >8,000 OOA participants. Here, we integrated data from two ongoing studies to conduct genetic analyses of adult-onset psychiatric disorders in the OOA population. The Amish Connectome Project (ACP) recruited individuals from OOA families in which at least two probands were affected with a severe mental illness^28^. Diagnoses with mental illnesses were obtained via the Structured Clinical Interview for DSM-IV or V (SCID)^75^. The Amish Wellness Study (AWS) recruited individuals from OOA families independent of any disease and surveyed a broad range of health-related phenotypes^76^. AWS collected primarily well individuals, and the rate of severe mental illnesses is expected to be low. Mood symptoms were assessed via the Patient Health Questionnaire-2 (PHQ-2)^77^. AWS participants who reported no current or lifetime history of mood symptoms were considered unaffected. AWS participants who reported mood symptoms or with missing PHQ-2 data were excluded from the analysis. All studies were approved by the Institutional Review Board at the University of Maryland Baltimore. Informed consent was obtained from each study participant.

### Exome sequencing.

Exome sequencing was performed using genomic DNA derived from the blood of ACP and AWS participants through a collaboration with the Regeneron Genetics Center (RGC). Exome sequencing of the AWS cohort has been described previously^24^. Exome sequencing of the ACP cohort was performed in parallel using an identical pipeline. Briefly, exome capture was performed using a slightly modified version of the xGen reagent (Integrated DNA Technologies). Sequencing was performed on the Illumina NovaSeq 6000 platform using paired-end 75 bp reads. Data processing was performed using a cloud-based pipeline implemented with DNAnexus, including sample de-multiplexing, alignment to the GRCh38 reference sequence, post-alignment BAM processing (e.g., marking of duplicate reads and other read mapping evaluation), and variant calling.

### Genetic association studies.

We computed associations of variants with MDD and BD affection status using generalized linear mixed models implemented with GMMAT^78^. We controlled for covariates of sex, age, and genetic relatedness, using a Balding-Nichols empirical kinship matrix computed from exome sequences with emmax-kin^79^. For the analysis of bipolar disorder, individuals with a diagnosis with bipolar disorder, type 1 were treated as cases, and individuals with no mental illness were treated as controls. All others were excluded from the analysis. An equivalent model was fit for cases with major depressive disorder. We did not test associations for schizophrenia spectrum disorders or other diagnoses because too few participants had these diagnoses.

### Variant filtering.

We annotated polymorphic variants in our exome sequencing dataset using the Ensembl Variant Effect Predictor^80^, as well as an in-house database of OOA-specific variant characteristics^81^. We defined high-quality OOA-enriched protein-coding single-nucleotide variants based on the following criteria: (i) MODERATE or HIGH impact (i.e., missense, splice site, and protein-trunacting variants) to a canonical transcript model; (ii) Combined Annotation Dependent Depletion (CADD)^82^ Phred score > 10; (iii) global minor allele frequency < 0.001 in both the gnomAD exomes^83^ and 1000 Genomes Phase 3^84^ datasets; (iv) OOA-specific minor allele frequency >1% and <10%; and (v) a Mendelian error rate of 0 (plink –mendel)^85^ in the AWS dataset.

### Prioritization of variants based prior associations with neuropsychiatric traits.

Our analysis focused primarily on OOA-enriched variants in established risk genes for psychiatric and neurodevelopmental disorders, as defined by the National Advisory Mental Health Council Workgroup on Genomics (https://www.nimh.nih.gov/research/priority-research-areas/genomics-research). In addition, we considered evidence for each gene’s association with psychiatric traits based on other types of genetic and genomic analysis, including GWAS of common variants, transcriptomic studies of post-mortem human brain tissue, and systems biology approaches. In total, we considered 30 gene lists from these prior studies (**Table S3**). For each gene list, we looked up which genes overlapped associations from our OOA cohort. We tested whether this overlap was greater than expected by chance using Fisher’s exact tests. In addition, we applied a network biology approach as part of our strategy to prioritize specific variants for follow-up, leveraging protein-protein interactions from the STRING database (v12)^86^. Over-representation for interactions was evaluated by Fisher’s exact tests and confirmed by edge permutations, as we have described previously^28^. To prioritize specific genes in a network context, we used igraph^87^ to compute each gene’s eigen-centrality in a STRING sub-graph composed of interactions between a gene set of interest and OOA-specific risk genes. The version of this analysis shown in Fig. S2 utilized targets of FMRP, which were the most strongly over-represented for interactions with OOA-specific risk genes. Similar results were obtained for other enriched gene lists.

### Genotype-first callback study of SETD1A P596L carriers and non-carriers to assess cognitive domains and symptom scales.

Leveraging existing genotypes together with pedigree structures from the Amish Genealogy Database^88^, we recruited probands who were known SETD1A P596L homozygotes and their first-degree relatives, as well as children for whom both parents were known to be heterozygotes. In families of these types, the genotypes of siblings in the same generation are expected to follow a Mendelian ratio of ¼ homozygotes, ½ heterozygotes, and ¼ non-carriers. SETD1A P596L genotypes were ascertained by Sanger sequencing of a PCR amplicon spanning the SETD1A P596L region, confirming 100% of genotypes for known carriers and non-carriers and establishing the genotypes of family members who had not enrolled in previous studies. Participants were interviewed in their homes to assess quantitative cognitive traits and schizophrenia-related symptoms, independent of diagnosis. Cognitive traits were assessed with the Brief Assessment of Cognition in Schizophrenia (BACS)^31^, which consists of six subtests in distinct cognitive domains. Although originally designed to assess cognition in SCZ, the BACS has also been validated to assess cognitive differences in individuals with BD and MDD^30,89^. Quantitative symptom scales were assessed with the Positive and Negative Syndrome Scale (PANSS)^33^, which consists of subtests for positive symptoms, negative symptoms, and general psychopathology.

### Cell lines and reprogramming to iPSCs.

Blood samples from two OOA homozygous for the SETD1A P596L variant (donor 669, male; donor 667, female) and one OOA non-carrier (donor 281, female) were collected in BD Vacutainer CPT tubes (BD Biosciences, CA). Peripheral blood mononuclear cells (PBMCs) were isolated via a ficoll gradient, resuspended in CryoStor CS10 freezing medium (StemCell Technologies, Vancouver, Canada) in cryovials, and stored in liquid nitrogen. Reprogramming of PBMCs into iPSCs was performed as previously described^90^. Briefly, reprogramming was performed by the National Heart Lung and Blood Institute (NHLBI-NIH) iPSC Core using the CytoTune-iPS 2.0 Sendai Reprogramming kit (Thermo Fisher Scientific, MA). iPSCs were characterized by examining the following factors: a) growth properties, b) sterility, c) absence of mycoplasma contamination, d) karyotype by spectral karyotyping (Cytogenetics & Microscopy Core, NHGRI, NIH), identity test (Fluidigm SNP Trace Panel, and expression of pluripotency markers (by FACS and/or immunocytochemistry). These iPSCs were produced as part of a broader iPSC resource from Amish and Mennonite families with mental illnesses. Detailed quality control metrics for this resource have been reported previously^90^. As an additional control, we also studied WA09 (“H9”) human embryonic stem cells (hESCs; WiCell, Madison, WI), which do not carry the SETD1A P596L variant.

### Cell culture and neural induction.

iPSCs and hESCs were maintained in Stemflex feeder-free medium (STEMCELL technologies) in a humidified incubator at 37 °C, 5% CO_2,_ and 20% O_2_.. CellAdhere^™^ Laminin-521, a defined xeno-free cell culture matrix, was used to maintain the cells in feeder-free conditions Cells were dissociated and passaged as aggregates using ReLeSR^™^ (STEMCELL technologies) without manual selection or scraping. iPSC/ESC were differentiated into neural progenitor cells (NPCs) using the STEMdiff^™^ SMADi Neural Induction Kit via embryoid bodies (EB) (STEMCELL technologies). Single EBs consisting of approximately 10,000 cells were generated in each microwell (800 mM diameter) of an AggreWell^™^800 plate (STEMCELL technologies), corresponding to ~300 EBs per well. Daily partial medium changes were performed until day 5, at which point the EBs were replated on Poly-L-Ornithine/Laminin-coated cultureware. The replated EBs were inspected daily for rosette formation characteristics, and allowed to grow to day 12 with daily medium change. Neural rosettes were selected on day 12, and the selected rosettes were replated on Poly-L-Ornithine/Laminin-coated plates and grown until 80% confluent (approximately 18–20 days). NPCs were passaged and maintained using STEMdiff^™^ Neural Progenitor Medium (STEMCELL technologies).

### DNA damage response assay.

To assess DNA double-stranded breaks (DDBs), phosphorylation of the Ser-139 residue of the histone variant H2AX, forming γH2AX, was used as a marker^34^. hiPSCs treated with hydroxyurea were harvested as described above. Cells were washed in PBS twice and fixed in 4% paraformaldehyde (PFA) at 37°C for 10 min. The fixed cells were immediately chilled on ice and washed three times with PBS-BSA (0.5% bovine serum albumin in PBS). The cells were then permeabilized with 70% ethanol in distilled water and stored at 4°C overnight. The cells were subsequently washed three times in PBS-BSA, and incubated in a 1/500 dilution of a polyclonal antibody against γH2AX (SIG-H5912) overnight at 4°C. The cells were washed three times with PBS-BSA and incubated in a 1/200 dilution of an Alexa 488-conjugated, anti-rabbit. Cells were washed free of secondary antibody and analyzed with a flow cytometer with excitation and emission set at 488 nm and 530 ± 20 nm, respectively.

### Cell proliferation.

Cell proliferation was determined using the EdU Staining Proliferation Kit (Abcam). Briefly, cells in the logarithmic phase were incubated for two hours in fresh medium supplemented with 10 mM of the modified thymidine analogue 5-ethynyl-2′-deoxyuridine (EdU). Incorporation of the EdU into the newly synthesized DNA was subsequently detected by a fluorescent azide through a Cu(I)-catalyzed cycloaddition reaction (“click” chemistry) and quantified using flow cytometry.

### Immunocytochemistry.

Cells were first washed twice with PBS, then fixed at the indicated time points by incubation in 4% paraformaldehyde (PFA) in PBS for 20 minutes at 4°C, followed by three washes for five minutes each in PBS. Cells were then permeabilized with 0.5% Triton X-100 in PBS for 20 minutes. Next, cells were washed twice for five minutes with PBS, blocked with 1% BSA in PBS for 1 hour at room temperature, and incubated overnight at 4°C on a rocking plate with the primary antibody diluted in PBS and 1% BSA. Primary antibodies include: SOX2 (1:200 dilution, R&D Systems, Catalog # AF2018); ZO-1/TJP1-FITC (1:250 dilution, INVITROGEN, Catalog # 339111); SOX1 (1:250 dilution, BD Biosciences, Catalog # 560749); NES-Alexa488 (1:100 dilution, STEMCELL Technologies, Catalog # 60091AD). After washing three times with PBS, cells were incubated with species-appropriate secondary antibodies conjugated to Alexa Fluorophores for 1 hour at room temperature in the dark. Secondary antibodies include: Donkey anti-goat Alexa 594 (1:500 dilution, INVITROGEN, Catalog # A11058); donkey anti-mouse Alexa 594 (1:500 dilution, INVITROGEN, Catalog # A21203). Coverslips were mounted and nuclei were counterstained using VECTASHIELD antifade mounting medium with DAPI (vector labs, catalog # H-1200–10).

### Image analysis: rosette counting, area measurements, neurite morphology, and soma size.

Image analysis was performed using ImageJ^91^. All analyses were conducted blinded to genotype and treatment. Neuroepithelial rosettes were identified by central ZO-1 localization with circular or rosette-like apical/basal arrangement of SOX2+ cells. The total number of rosettes and area of expansion were manually annotated and quantified. NPC and neurite morphology were quantified in Nestin-stained images. Cell bodies were segmented based on DAPI and soma shape and size thresholds. Neurites were manually counted and measured, and quantified to average neurite length and total number of neurites per cell. Soma cross-sectional area was extracted from the segmented cell body masks.

### β-galactosidase staining.

β-galactosidase activity was assessed using the senescence β-galactosidase staining kit (Cell Signaling Technology, catalog # 9860S) according to the manufacturer’s instructions. Briefly, 10x fixation solution, 10x staining solution, and X-gal were redissolved and diluted to 1x working concentrations. Cells were rinsed with PBS 1x and fixed in fixation solution for 15 minutes at room temperature. 1x staining solution, 100x solution A, 100x solution B, and 20mg/ml X-gal solution were combined while cells were being fixed to make the β-galactosidase staining solution. Cells were washed twice with PBS and immediately incubated in β-galactosidase staining solution overnight at 37°C in a dry incubator. The following day, the solution was aspirated, cells were washed 1x with PBS, and mounted with VECTASHIELD antifade mounting medium (vector labs, catalog # H-1000–10). β-galactosidase–positive cells were identified by the characteristic blue cytoplasmic stain and quantified manually from brightfield images. Results were expressed as the percentage of β-gal–positive cells among total cells per field. Matched X-gal and soma size measurements were also used to determine a soma size threshold at which most cells are X-gal-positive (i.e., senescent). To determine this threshold, we fit a logistic regression data to predict X-gal staining in the matched data, then applied this model to additional images for which soma size measurements were obtained but not X-gal.

### SETD1A overexpression.

Cells were transfected with full-length SETD1A mRNA to rescue proliferation deficiency associated with SETD1A P596L. The Ribomax Large Scale RNA Production System-t7 (Promega) was used to *in vitro* transcribe full length SETD1A mRNA from a cDNA template. The Ribo M7g Cap Analog (Promega) was used to cap the transcript. The Monarch RNA Cleanup Kit (New England Bio Labs) was used to clean up the in vitro-transcribed mRNA. Purified mRNA was quantified using the qubit RNA BR kit (Invitrogen). 250 ng of purified SETD1A mRNA was co-delivered during the time of RNP transfection. Following transfection, cell number and proliferation were determined as described above.

### KDM5 inhibitor treatment.

To pharmacologically modulate H3K4 demethylase activity, cultures were treated with the KDM5 inhibitor CPI-455 HCl (Selleckchem, catalog no. S8287). A 10 mM stock solution was prepared in DMSO and stored at −20°C. Cells were treated with CPI-455 at a final concentration of 10 or 20 μM starting at DIV0 and maintained for the duration of the protocol. Control cultures received an equivalent volume of DMSO.

### mRNA sequencing.

Total RNA was extracted from iPSC–derived neural cultures at defined stages of neural induction and at ~80% confluency after each NPC passage (DIV: 0, 2, 8, 12, 20, 23 (p1), 28 (p2), 33 (p3), 38 (p4), 44 (p5), 50 (p6)) using the RNeasy Mini Kit (QIAGEN). RNA integrity was assessed on an Agilent 4200 TapeStation system (Agilent) to confirm RNA integrity numbers (RINs ≥ 8.7). Strand-specific mRNA libraries were prepared and sequenced on a NovaSeq 6000 sequencer (Illumina) to a depth of ~50 million raw reads per library. Library preparation and sequencing were performed by the MD Genomics core at the Institute for Genome Sciences (IGS) of the University of Maryland School of Medicine. Transcript abundance was quantified using Kallisto^92^. Transcript counts were summarized to gene level, normalized to counts per million via edgeR^93^ and log transformed for farther analysis.

### Single-cell RNA/ATAC multi-ome sequencing.

To reduce artifacts related to cell cycle asynchrony, iPSCs were synchronized in G2/M by treatment with 25 ng/mL nocodazole (Sigma-Aldrich) for 16 hr. For release into G1, nocodazole was removed, cells were washed twice with DMEM/F12, and resuspended in maintenance medium. Neural induction was performed as described above, and nuclei were isolated from iPSC-derived neural cultures at DIV 0, 2, 8, and 20 and prepared according to the 10x Genomics validated protocol for adherent cells (CG000365). Nuclei concentration and quality were assessed using MoxiGO II (Orflo Technologies). Nuclei pooled from one SETD1A P596L line (669A) and one non-carrier lines (281B) were loaded into a Chromium X Controller (10x Genomics) using the Chromium Single Cell Multiome ATAC + Gene Expression kit (10x Genomics). Library preparation and sequencing on a NovaSeq 6000 sequencer were performed by the MD Genomics core.

### scMultiome data processing.

Raw scMultiome sequencing data were processed using Cell Ranger ARC (10x Genomics) and aligned to GRCh38 reference genome. Gene expression count matrices and fragment files for chromatin accessibility for each sample were aggregated using the Cell Ranger ARC aggr function. Genotype-based demultiplexing was performed with demuxlet^94^ using genotype VCF files from exome sequencing of donors and the 10x Genomics gene expression BAM files generated by Cell Ranger ARC. Subsequent data processing steps were performed in R using Seurat v4^95^ and Signac v2^96^. For the RNA modality, cells were retained if they expressed between 5,000–25,000 unique molecules and had fewer than 20% of reads mapping to mitochondrial genes. For the ATAC modality, nuclei were filtered by requiring <50,000 total fragments per cell, a transcription start site (TSS) enrichment score > 1, and a nucleosome signal ≤ 1.5. Raw RNA counts were log normalized with a scale factor of 10,000 in Seurat, and the top 3000 highly variable genes were retained for downstream analyses. ATAC data were processed by constructing a peak-by-cell matrix using MACS2, applying term frequency–inverse document frequency (TF–IDF) normalization, and performing singular value decomposition (SVD) on the resulting matrix.

### Integration, dimensionality reduction, and cell type annotation.

To integrate data across donors and time points, Seurat’s anchor-based integration was used. Briefly, RNA samples were first integrated across each timepoint using 3,000 highly-variable features to minimize batch effects, then new integration anchors were calculated across genotypes to harmonize RNA expression values. For the ATAC data, we first compute latent semantic indexing (LSI) embeddings for each cell. We then integrated across all donors and timepoints using reciprocal latent semantic indexing (rLSI) projection from the first 40 dimensions. Dimensionality reduction was performed by principal component analysis (PCA) on normalized, integrated gene expression values using the top 3,000 highly variable genes and by LSI on the integrated ATAC count matrix. A weighted nearest neighbors (WNN) graph was constructed using 50 PCs and 40 LSI components and used to make the UMAP for clustering and visualization. Cell types were assigned based on the expression of established marker genes and confirmed by correlation with published single-cell reference datasets from developing neocortical cell types^97^. In addition, cells were annotated to cell cycle state using Seurat’s cell-cycle scoring pipeline. Briefly, we applied CellCycleScoring with curated S-phase and G2/M-phase gene sets (Seurat cc.genes.updated.2019) to compute per-cell S.Score and G2M.Score. Cells were assigned to discrete phases (G1, S, or G2M) based on the relative magnitudes of these scores.

### Principal component analysis and transfer learning.

Principal component analysis (PCA) was performed on bulk gene expression data to assess dominant sources of variance across samples. PCA was computed using the prcomp function in R with default parameters. The proportion of variance explained by each principal component was calculated as the squared singular values divided by their sum and used to quantify the contribution of individual components to total expression variability. Principal components from our data were projected into reference datasets^41,98^ using projectR^40^ to characterize their associations with neurodevelopmental cell types and trajectories.

### Pseudotemporal trajectories.

Cells were ordered in pseudotime using Monocle3^99^. Briefly, the Seurat object was converted to a Monocle3 cell data set using SeuratWrappers and preprocessed using the standard Monocle3 pipeline. Trajectories were learned on the weighted nearest neighbors UMAP computed in Seurat. The starting trajectory root was defined as the principal graph node most frequently associated with cells in pluripotency, computed with igraph^87^.

### Differential gene expression analysis.

Differential gene expression analyses were performed to detect effects of P596L genotype using the limma voom pipeline^100^ to fit linear models and test post-hoc contrasts at each time point. Genes were considered differentially expressed if they had an FDR ≤ 0.05 and |logFC| ≥ 1. Gene set enrichment analyses of differentially expressed genes were performed with DAVID^101^ using the full set of expressed genes as background. Gene Ontology (GO) biological process, molecular function, and cellular component categories were tested, along with KEGG and Reactome pathways, using default settings and Benjamini–Hochberg correction.

### Gene co-expression modules.

Gene co-expression modules were calculated from scRNA-seq data as previously described^47^. Briefly, read counts were imputed to restore correlation structure using knn-smoothing (k = 15, d= 30). We then performed k-means clustering (k = 50) to identify modules of co-expresed genes. Genes with weak correlations to the module centroid were removed (r < 0.3). Modules with strongly correlated centroids were merged by average-linkage hierarchical clustering using 1 – Pearson’s r as a distance metric, with a cut height = 0.15. To annotate co-expression modules to known functional categories and pathways, we performed gene set enrichment analysis using the fGSEA R package^102^. Within each module, genes were ranked by module membership (kME), the strength of the Pearson correlation between each gene’s expression and the module centroid. Enrichment was assessed against curated gene sets, including MSigDB hallmark and GO collections (MSigDB). Enrichment scores and adjusted P values were computed using 10,000 permutations per gene set.

### Gene regulatory networks.

We reconstructed a gene regulatory network (GRN) model using our scMultiome data, similar to our prior work^47^. Briefly, model reconstruction began with a physical model of transcription factor binding sites within accessible chromatin peak, using motifs from JASPAR2020 within Signac^96^ and motif-to-TF mappings from the MotifDb R package. The resulting TF occupancy site predictions were then mapped to potential target genes, where proximal peaks were mapped to transcription start sites (TSSs) within 2 kb and distal peaks were mapped to TSSs via co-accessibility, computed with Cicero^46^. Next, we applied a random forest regression framework (GENIE3^48^) to identify a set of TFs with binding sites in each gene’s regulatory elements that together predict that genes expression variability across cells in our scMultuome dataset. We retained the top 200,000 TF-gene interactions, and each TF-gene interaction was assigned a regulatory type (activating vs repressing) by the sign of the Pearson correlation between the expression of the TF and the target gene across all cells. To identify master regulators of gene co-expression modules, we tested for overrepresentation of a TF’s predicted targets within a given module using Fisher’s exact tests, testing activating and repressing targets separately. We validated the computationally-derived E2F7 regulon using E2F7 ChIP–seq data from ENCODE^42,103^. E2F7 peak regions (ENCFF938ZPZ) were annotated to the nearest TSS (±2 kb) using ChIPseeker^104^.

### Differential abundance/cell-type composition.

Differential abundance was tested as genotype-associated differences in cellular composition using aggregated count-based binomial generalized linear models. For each timepoint, counts were summarized as k cells for a given cell-type out of N total cells per genotype, and a binomial generalized linear model was fit to test for a genotype effect on the proportion of each cell-type at each time point.

### Statistical analyses.

Quantitative data on cell phenotypes were fit to linear and generalized linear mixed-effects models, using the lmer and glmer R packages, including the following phenotypes and link functions: EdU incorporation (binomial), rosette number (linear), neurite length (linear), neurite counts (poisson), soma area (linear), and X-gal expression (binomial). We modeled fixed effects of SETD1A P596L genotype and perturbations (CPI-455, SETD1A overexpression), as well as random effects of cell line and time point. Statistical methods for genetic and genomic data are described in the preceding sections.

## Supplementary Material

Fig S1 to S10

Tables S1 to S14

This is a list of supplementary files associated with this preprint. Click to download.

- LeaseEtAlSETD1Amssupplementfigures.pdf
- SupplTables20240325.xlsx

## Data Availability

Complete summary-level data from exome sequencing and phenotypic data from the Amish Connectome Project and Amish Wellness Study are provided in the supplementary tables. Individual-level genotypic and phenotypic data will be provided upon reasonable request to the investigators. Genotypic and phenotypic data from the genotype-first callback study of SETD1A P596L carriers and non-carriers, as well as RNA-seq and scMultiome data, have been deposited in the NIMH Data Archive (10.15154/8dm1-f069). A web portal providing dynamic visualizations of the transcriptomic data is available on NeMO Analytics (https://nemoanalytics.org/p?l=5c559012). Induced pluripotent stem cell lines have been deposited in the NIMH Cell and DNA Repository. Microscopy images have been deposited at Bioimage Archive (S-BIAD3289). Custom code used in the analysis have been deposited in GitHub (https://github.com/seth-ament/SETD1A_P596L). All other data are available in the main text or the supplementary materials.

## References

[1] Baselmans B. M. L., Yengo L., van Rheenen W. & Wray N. R. Risk in Relatives, Heritability, SNP-Based Heritability, and Genetic Correlations in Psychiatric Disorders: A Review. Biol. Psychiatry 89, 11–19 (2021). 32736793 10.1016/j.biopsych.2020.05.034

[2] Glahn D. C. Rediscovering the value of families for psychiatric genetics research. Mol. Psychiatry 24, (2019). 10.1038/s41380-018-0073-x PMC7028329 29955165

[3] Singh T. Rare coding variants in ten genes confer substantial risk for schizophrenia. Nature doi: 10.1038/s41586-022-04556-w (2022) doi:10.1101/2020.09.18.20192815. PMC9805802 35396579

[4] Satterstrom F. K. Large-Scale Exome Sequencing Study Implicates Both Developmental and Functional Changes in the Neurobiology of Autism. Cell 180, 568–584.e23 (2020). 31981491 10.1016/j.cell.2019.12.036 PMC7250485

[5] Singh T. Rare loss-of-function variants in SETD1A are associated with schizophrenia and developmental disorders. Nat. Neurosci. 19, 571–577 (2016). 26974950 10.1038/nn.4267 PMC6689268

[6] Kummeling J. Characterization of SETD1A haploinsufficiency in humans and Drosophila defines a novel neurodevelopmental syndrome. Mol. Psychiatry https://doi.org/10.1038/s41380-020-0725-5 (2020) doi:10.1038/s41380-020-0725-5. 32346159

[7] Takata A. Loss-of-Function Variants in Schizophrenia Risk and SETD1A as a Candidate Susceptibility Gene. Neuron 82, 773–780 (2014). 24853937 10.1016/j.neuron.2014.04.043 PMC4387883

[8] Su X. Mutations of schizophrenia risk gene SETD1A dysregulate synaptic function in human neurons. Mol. Psychiatry 30, 5680–5693 (2025). 40962831 10.1038/s41380-025-03246-z PMC12602330

[9] Mukai J. Recapitulation and Reversal of Schizophrenia-Related Phenotypes in Setd1a-Deficient Mice. Neuron 104, 471–487.e12 (2019). 31606247 10.1016/j.neuron.2019.09.014 PMC7010348

[10] Hamm J. P., Shymkiv Y., Mukai J., Gogos J. A. & Yuste R. Aberrant Cortical Ensembles and Schizophrenia-like Sensory Phenotypes in Setd1a+/− Mice. Biol. Psychiatry 88, 215–223 (2020). 32143831 10.1016/j.biopsych.2020.01.004 PMC7363535

[11] Clifton N. E. Setd1a Loss-of-function Disrupts Epigenetic Regulation of Ribosomal Genes via Altered DNA Methylation. Schizophr. Bull. https://doi.org/10.1093/schbul/sbaf091 (2025) doi:10.1093/schbul/sbaf091. PMC12996923 40500874

[12] Nagahama K. Setd1a Insufficiency in Mice Attenuates Excitatory Synaptic Function and Recapitulates Schizophrenia-Related Behavioral Abnormalities. Cell Rep. 32, (2020). 10.1016/j.celrep.2020.108126 32937141

[13] Clifton N. E. Developmental disruption to the cortical transcriptome and synaptosome in a model of SETD1A loss-of-function. Hum. Mol. Genet. 31, 3095–3106 (2022). 35531971 10.1093/hmg/ddac105 PMC9476630

[14] Zhao T. Epigenetic maintenance of adult neural stem cell quiescence in the mouse hippocampus via Setd1a. Nat. Commun. 15, (2024). 10.1038/s41467-024-50010-y PMC11227589 38971831

[15] Sawada T. SETD1A regulates psychiatric gene networks involved in genomic stability and synaptic function in rare and sporadic schizophrenia. Nat. Commun. 17, 461 (2025). 41422157 10.1038/s41467-025-67154-0 PMC12800067

[16] Wang S. Loss-of-function variants in the schizophrenia risk gene SETD1A alter neuronal network activity in human neurons through the cAMP/PKA pathway. Cell Rep. 39, (2022). 10.1016/j.celrep.2022.110790 PMC7615788 35508131

[17] Hupalo S. Altered thalamo-prefrontal synchrony dynamics during spatial working memory task performance in a SETD1A loss-of-function mouse model of schizophrenia predisposition. bioRxiv https://doi.org/10.64898/2026.01.29.702577 (2026) doi:10.64898/2026.01.29.702577. 42608525

[18] Sun Z. Genomic and Transcriptomic Signatures of SETD1A Disruption in Human Excitatory Neuron Development and Psychiatric Disease Risk. bioRxiv https://doi.org/10.1101/2025.03.26.645419 (2025) doi:10.1101/2025.03.26.645419.

[19] Toyoda S. Schizophrenia-related Xpo7 haploinsufficiency leads to behavioral and nuclear transport pathologies. EMBO Rep. 26, 948–981 (2025). 39774335 10.1038/s44319-024-00362-9 PMC11850608

[20] Nakamura T. & Takata A. The molecular pathology of schizophrenia: an overview of existing knowledge and new directions for future research. Mol. Psychiatry 28, 1868–1889 (2023). 36878965 10.1038/s41380-023-02005-2 PMC10575785

[21] Curtis D. Clinical features of UK Biobank subjects carrying protein-truncating variants in genes implicated in schizophrenia pathogenesis. Psychiatr. Genet. 32, 156–161 (2022). 35749744 10.1097/YPG.0000000000000318

[22] Hou L. Amish revisited: next-generation sequencing studies of psychiatric disorders among the Plain people. Trends Genet. 29, 412–8 (2013). 23422049 10.1016/j.tig.2013.01.007 PMC3941079

[23] Pollin T. I. A null mutation in human APOC3 confers a favorable plasma lipid profile and apparent cardioprotection. Science 322, 1702–5 (2008). 19074352 10.1126/science.1161524 PMC2673993

[24] Montasser M. E. Genetic and functional evidence links a missense variant in B4GALT1 to lower LDL and fibrinogen. Science 374, 1221–1227 (2021). 34855475 10.1126/science.abe0348

[25] Kurki M. I. FinnGen provides genetic insights from a well-phenotyped isolated population. Nature 2023 613:7944 613, 508–518 (2023). 10.1038/s41586-022-05473-8 PMC9849126 36653562

[26] Gudbjartsson D. F. Large-scale whole-genome sequencing of the Icelandic population. Nature Genetics 2015 47:5 47, 435–444 (2015). 25807286 10.1038/ng.3247

[27] Strauss K. A. A population-based study of KCNH7 p.Arg394His and bipolar spectrum disorder. Hum. Mol. Genet. https://doi.org/10.1093/hmg/ddu335 (2014) doi:10.1093/hmg/ddu335. PMC4222358 24986916

[28] Humphries E. M. Genome-wide significant risk loci for mood disorders in the Old Order Amish founder population. Molecular Psychiatry 2023 1–10 (2023) doi:10.1038/s41380-023-02014-1. 36882501 PMC10483025

[29] Hasin N. Rare variants implicate NMDA receptor signaling and cerebellar gene networks in risk for bipolar disorder. Mol. Psychiatry https://doi.org/10.1038/S41380-022-01609-4 (2022) doi:10.1038/S41380022-01609-4. 35546635

[30] Cholet J. Using the Brief Assessment of Cognition in Schizophrenia (BACS) to assess cognitive impairment in older patients with schizophrenia and bipolar disorder. Bipolar Disord. 16, 326–336 (2014). 24383665 10.1111/bdi.12171

[31] Keefe R. S. E. Norms and standardization of the Brief Assessment of Cognition in Schizophrenia (BACS). Schizophr. Res. 102, 108–115 (2008). 18495435 10.1016/j.schres.2008.03.024

[32] Daneluzzo E. PANSS factors and scores in schizophrenic and bipolar disorders during an index acute episode: a further analysis of the cognitive component. Schizophr. Res. 56, 129–136 (2002). 12084427 10.1016/s0920-9964(01)00277-8

[33] Kay S. R., Fiszbein A. & Opler L. A. The positive and negative syndrome scale (PANSS) for schizophrenia. Schizophr. Bull. 13, 261–276 (1987). 3616518 10.1093/schbul/13.2.261

[34] Mah L. J., El-Osta A. & Karagiannis T. C. gammaH2AX: a sensitive molecular marker of DNA damage and repair. Leukemia 24, 679–686 (2010). 20130602 10.1038/leu.2010.6

[35] Tajima K. SETD1A protects from senescence through regulation of the mitotic gene expression program. Nat. Commun. 10, (2019). 10.1038/s41467-019-10786-w PMC6599037 31253781

[36] Tajima K. SETD1A modulates cell cycle progression through a miRNA network that regulates p53 target genes. Nat. Commun. 6, (2015). 10.1038/ncomms9257 PMC4667427 26394836

[37] Bledau A. S. The H3K4 methyltransferase Setd1a is first required at the epiblast stage, whereas Setd1b becomes essential after gastrulation. Development 141, 1022–1035 (2014). 24550110 10.1242/dev.098152

[38] Pollen A. A. & Kriegstein A. R. Diversity and Evolution of Human Cortical Progenitor Cell Types. Neocortical Neurogenesis in Development and Evolution 19–39 (2023) doi:10.1002/9781119860914.ch2.

[39] Micali N. Variation of Human Neural Stem Cells Generating Organizer States In Vitro before Committing to Cortical Excitatory or Inhibitory Neuronal Fates. Cell Rep. 31, (2020). 10.1016/j.celrep.2020.107599 PMC7357345 32375049

[40] Sharma G., Colantuoni C., Goff L. A., Fertig E. J. & Stein-O’Brien G. projectR: an R/Bioconductor package for transfer learning via PCA, NMF, correlation and clustering. Bioinformatics 36, 3592–3593 (2020). 32167521 10.1093/bioinformatics/btaa183 PMC7267840

[41] van de Leemput J. CORTECON: a temporal transcriptome analysis of in vitro human cerebral cortex development from human embryonic stem cells. Neuron 83, 51–68 (2014). 24991954 10.1016/j.neuron.2014.05.013

[42] Trevino A. E. Chromatin and gene-regulatory dynamics of the developing human cerebral cortex at single-cell resolution. Cell https://doi.org/10.1016/J.CELL.2021.07.039 (2021) doi:10.1016/J.CELL.2021.07.039. 34390642

[43] Sonthalia S. A Curated Compendium of Transcriptomic Data for the Exploration of Neocortical Development. bioRxiv https://doi.org/10.1101/2024.02.26.581612 (2024) doi:10.1101/2024.02.26.581612. PMC13061640 41882195

[44] Orvis J. gEAR: Gene Expression Analysis Resource portal for community-driven, multi-omic data exploration. Nature Methods 2021 18:8 18, 843–844 (2021). 34172972 10.1038/s41592-021-01200-9 PMC8996439

[45] Zhang L. Cellular senescence: a key therapeutic target in aging and diseases. J. Clin. Invest. 132, e158450 (2022). 35912854 10.1172/JCI158450 PMC9337830

[46] Pliner H. A. Cicero Predicts cis-Regulatory DNA Interactions from Single-Cell Chromatin Accessibility Data. Mol. Cell 71, 858–871.e8 (2018). 30078726 10.1016/j.molcel.2018.06.044 PMC6582963

[47] Ament S. A. A single-cell genomic atlas for maturation of the human cerebellum during early childhood. Sci. Transl. Med. 15, (2023). 10.1126/scitranslmed.ade1283 37824600

[48] Huynh-Thu V. A., Irrthum A., Wehenkel L. & Geurts P. Inferring regulatory networks from expression data using tree-based methods. PLoS One 5, (2010). 10.1371/journal.pone.0012776 PMC2946910 20927193

[49] Sadasivam S. & DeCaprio J. A. The DREAM complex: master coordinator of cell cycle-dependent gene expression. Nat. Rev. Cancer 13, 585–595 (2013). 23842645 10.1038/nrc3556 PMC3986830

[50] Lisek M., Przybyszewski O., Zylinska L., Guo F. & Boczek T. The Role of MEF2 Transcription Factor Family in Neuronal Survival and Degeneration. Int. J. Mol. Sci. 24, (2023). 10.3390/ijms24043120 PMC9963821 36834528

[51] Sarachana T. & Hu V. W. Genome-wide identification of transcriptional targets of RORA reveals direct regulation of multiple genes associated with autism spectrum disorder. Mol. Autism 4, (2013). 10.1186/2040-2392-4-14 PMC3665583 23697635

[52] Park S. H. E., Kulkarni A. & Konopka G. FOXP1 orchestrates neurogenesis in human cortical basal radial glial cells. PLoS Biol. 21, (2023). 10.1371/journal.pbio.3001852 PMC10431666 37540706

[53] Xia H. Building a schizophrenia genetic network: transcription factor 4 regulates genes involved in neuronal development and schizophrenia risk. Hum. Mol. Genet. 27, 3246–3256 (2018). 29905862 10.1093/hmg/ddy222 PMC6354221

[54] Vinogradova M. An inhibitor of KDM5 demethylases reduces survival of drug-tolerant cancer cells. Nat. Chem. Biol. 12, 531–538 (2016). 27214401 10.1038/nchembio.2085

[55] Higgs M. R. Histone Methylation by SETD1A Protects Nascent DNA through the Nucleosome Chaperone Activity of FANCD2. Mol. Cell 71, 25 (2018). 29937342 10.1016/j.molcel.2018.05.018 PMC6039718

[56] Barch D. M. & Ceaser A. Cognition in Schizophrenia: Core Psychological and Neural Mechanisms. Trends Cogn. Sci. 16, 10.1016/j.tics.2011.11.015 (2011). PMC3860986 22169777

[57] Wang S. SETD1A Mediated H3K4 Methylation and Its Role in Neurodevelopmental and Neuropsychiatric Disorders. Front. Mol. Neurosci. 14, 772000 (2021). 34803610 10.3389/fnmol.2021.772000 PMC8595121

[58] Higgs M. R. Histone Methylation by SETD1A Protects Nascent DNA through the Nucleosome Chaperone Activity of FANCD2. Mol. Cell 71, 25–41.e6 (2018). 29937342 10.1016/j.molcel.2018.05.018 PMC6039718

[59] Hoshii T. A Non-catalytic Function of SETD1A Regulates Cyclin K and the DNA Damage Response. Cell 172, 1007–1021.e17 (2018). 29474905 10.1016/j.cell.2018.01.032 PMC6052445

[60] Paulsen B. Autism genes converge on asynchronous development of shared neuron classes. Nature 602, 268–273 (2022). 35110736 10.1038/s41586-021-04358-6 PMC8852827

[61] Carosso G. A. Precocious neuronal differentiation and disrupted oxygen responses in Kabuki syndrome. JCI Insight 4, (2019). 10.1172/jci.insight.129375 PMC6824316 31465303

[62] KMT2D-deficiency destabilizes lineage progression in immature neural progenitors - Search Results - PubMed. https://pubmed.ncbi.nlm.nih.gov/?term=KMT2Ddeficiency+destabilizes+lineage+progression+in+immature+neural+progenitors&sort=date.

[63] Ding S. CHD8 safeguards early neuroectoderm differentiation in human ESCs and protects from apoptosis during neurogenesis. Cell Death Dis. 12, (2021). 10.1038/s41419-021-04292-5 PMC8536677 34686651

[64] Lalli M. A., Avey D., Dougherty J. D., Milbrandt J. & Mitra R. D. High-throughput single-cell functional elucidation of neurodevelopmental disease-associated genes reveals convergent mechanisms altering neuronal differentiation. Genome Res. 30, 1317–1331 (2020). 32887689 10.1101/gr.262295.120 PMC7545139

[65] Brooks P. J., Cheng T. F. & Cooper L. Do all of the neurologic diseases in patients with DNA repair gene mutations result from the accumulation of DNA damage? DNA Repair (Amst). 7, 834 (2008). 18339586 10.1016/j.dnarep.2008.01.017 PMC2408373

[66] Ribeiro J. H. DNA damage and repair: underlying mechanisms leading to microcephaly. Front. Cell Dev. Biol. 11, (2023). 10.3389/fcell.2023.1268565 PMC10597653 37881689

[67] Rayi A. & Hozayen S. Chromosome Instability Syndromes. The Agt Cytogenetics Laboratory Manual, Fourth Edition 653–685 (2022) doi:10.1002/9781119061199.ch13.

[68] Jourdon A., Fasching L., Scuderi S., Abyzov A. & Vaccarino F. M. The role of somatic mosaicism in brain disease. Curr. Opin. Genet. Dev. 65, 84 (2020). 32622340 10.1016/j.gde.2020.05.002 PMC7749073

[69] Antonijoan R. M. First-in-Human Randomized Trial to Assess Safety, Tolerability, Pharmacokinetics and Pharmacodynamics of the KDM1A Inhibitor Vafidemstat. CNS Drugs 35, 331–344 (2021). 33755924 10.1007/s40263-021-00797-x PMC7985749

[70] Ferrer M. REIMAGINE: A central nervous system basket trial showing safety and efficacy of vafidemstat on aggression in different psychiatric disorders. Psychiatry Clin. Neurosci. 79, 257–265 (2025). 39936839 10.1111/pcn.13800 PMC12047063

[71] Friedman J. I. CNTNAP2 gene dosage variation is associated with schizophrenia and epilepsy. Mol. Psychiatry 13, 261–266 (2008). 17646849 10.1038/sj.mp.4002049

[72] Detera-Wadleigh S. D. A resource of induced pluripotent stem cell (iPSC) lines including clinical, genomic, and cellular data from genetically isolated families with mood and psychotic disorders. Transl. Psychiatry 13, (2023). 10.1038/s41398-023-02641-w PMC10725500 38104115

[73] Hostetler J. A.. Amish society. 435 (1993).

[74] Strauss K. A. & Puffenberger E. G. Genetics, medicine, and the Plain people. Annu. Rev. Genomics Hum. Genet. 10, 513–536 (2009). 19630565 10.1146/annurev-genom-082908-150040

[75] First M. B., Williams J. B. W., Karg R. S. & Spitzer R. L. User’s Guide for the SCID-5-CV Structured Clinical Interview for DSM-5fi Disorders. (American Psychiatric Publishing, Inc., Washington, D.C., 2016).

[76] He S. Prevalence, control, and treatment of diabetes, hypertension, and high cholesterol in the Amish. BMJ Open Diabetes Res. Care 8, (2020). 10.1136/bmjdrc-2019-000912 PMC7449360 32843497

[77] Kroenke K., Spitzer R. L. & Williams J. B. W. The Patient Health Questionnaire-2: validity of a two-item depression screener. Med. Care 41, 1284–1292 (2003). 14583691 10.1097/01.MLR.0000093487.78664.3C

[78] H C. Control for Population Structure and Relatedness for Binary Traits in Genetic Association Studies via Logistic Mixed Models. Am. J. Hum. Genet. 98, 653–666 (2016). 27018471 10.1016/j.ajhg.2016.02.012 PMC4833218

[79] Kang H. M. Variance component model to account for sample structure in genome-wide association studies. Nat. Genet. 42, 348–54 (2010). 20208533 10.1038/ng.548 PMC3092069

[80] McLaren W. The Ensembl Variant Effect Predictor. Genome Biol. 17, (2016). 10.1186/s13059-016-0974-4 PMC4893825 27268795

[81] Perry J. A., Gaynor B. J., Mitchell B. D. & O’Connell J. R. An Omics Analysis Search and Information System (OASIS) for Enabling Biological Discovery in the Old Order Amish. bioRxiv 2021.05.02.442370 (2021) doi:10.1101/2021.05.02.442370.

[82] Rentzsch P., Witten D., Cooper G. M., Shendure J. & Kircher M. CADD: predicting the deleteriousness of variants throughout the human genome. Nucleic Acids Res. 47, D886–D894 (2019). 30371827 10.1093/nar/gky1016 PMC6323892

[83] Karczewski K. J. The mutational constraint spectrum quantified from variation in 141,456 humans. Nature 581, 434–443 (2020). 32461654 10.1038/s41586-020-2308-7 PMC7334197

[84] Auton A. A global reference for human genetic variation. Nature 526, 68–74 (2015). 26432245 10.1038/nature15393 PMC4750478

[85] Purcell S. PLINK: a tool set for whole-genome association and population-based linkage analyses. Am. J. Hum. Genet. 81, 559–75 (2007). 17701901 10.1086/519795 PMC1950838

[86] Szklarczyk D. STRING v11: protein–protein association networks with increased coverage, supporting functional discovery in genome-wide experimental datasets. Nucleic Acids Res. 47, D607–D613 (2019). 30476243 10.1093/nar/gky1131 PMC6323986

[87] Csardi G. & Nepusz T. The igraph software package for complex network research. InterJournal, Complex Systems http://www.necsi.edu/events/iccs6/papers/c1602a3c126ba822d0bc4293371c.pdf (2006).

[88] Agarwala R., Biesecker L. G. & Schӓffer A. A. Anabaptist genealogy database. Am. J. Med. Genet. C Semin. Med. Genet. 121C, 32–37 (2003). 12888984 10.1002/ajmg.c.20004

[89] Terachi S. Comparison of neurocognitive function in major depressive disorder, bipolar disorder, and schizophrenia in later life: A cross-sectional study of euthymic or remitted, non-demented patients using the Japanese version of the Brief Assessment of Cognition in Schizophrenia (BACS-J). Psychiatry Res. 254, 205–210 (2017). 28476012 10.1016/j.psychres.2017.04.058

[90] Detera-Wadleigh S. D. A resource of induced pluripotent stem cell (iPSC) lines including clinical, genomic, and cellular data from genetically isolated families with mood and psychotic disorders. Transl. Psychiatry 13, (2023). 10.1038/s41398-023-02641-w PMC10725500 38104115

[91] Schneider C. A., Rasband W. S. & Eliceiri K. W. NIH Image to ImageJ: 25 years of image analysis. Nat. Methods 9, 671–675 (2012). 22930834 10.1038/nmeth.2089 PMC5554542

[92] Bray N. L., Pimentel H., Melsted P. & Pachter L. Near-optimal probabilistic RNA-seq quantification. Nat. Biotechnol. 34, 525–527 (2016). 27043002 10.1038/nbt.3519

[93] Robinson M. D., McCarthy D. J. & Smyth G. K. edgeR: a Bioconductor package for differential expression analysis of digital gene expression data. Bioinformatics 26, 139–40 (2010). 19910308 10.1093/bioinformatics/btp616 PMC2796818

[94] Kang H. M. Multiplexed droplet single-cell RNA-sequencing using natural genetic variation. Nat. Biotechnol. 36, 89–94 (2018). 29227470 10.1038/nbt.4042 PMC5784859

[95] Butler A., Hoffman P., Smibert P., Papalexi E. & Satija R. Integrating single-cell transcriptomic data across different conditions, technologies, and species. Nat. Biotechnol. 36, 411–420 (2018). 29608179 10.1038/nbt.4096 PMC6700744

[96] Stuart T., Srivastava A., Lareau C. & Satija R. Multimodal single-cell chromatin analysis with Signac. bioRxiv 2020.11.09.373613 (2020) doi:10.1101/2020.11.09.373613.

[97] La Manno G. Molecular architecture of the developing mouse brain. Nature 596, 92–96 (2021). 34321664 10.1038/s41586-021-03775-x

[98] Li M. Integrative functional genomic analysis of human brain development and neuropsychiatric risks. Science (1979). 362, eaat7615 (2018). 10.1126/science.aat7615 PMC6413317 30545854

[99] Trapnell C. The dynamics and regulators of cell fate decisions are revealed by pseudotemporal ordering of single cells. Nat. Biotechnol. 32, 381–386 (2014). 24658644 10.1038/nbt.2859 PMC4122333

[100] Law C. W., Chen Y., Shi W. & Smyth G. K. Voom: Precision weights unlock linear model analysis tools for RNA-seq read counts. Genome Biol. 15, (2014). 10.1186/gb-2014-15-2-r29 PMC4053721 24485249

[101] Huang D. W., Sherman B. T. & Lempicki R. A. Systematic and integrative analysis of large gene lists using DAVID bioinformatics resources. Nat. Protoc. 4, 44–57 (2009). 19131956 10.1038/nprot.2008.211

[102] Korotkevich G. Fast gene set enrichment analysis. bioRxiv 060012 (2021) doi:10.1101/060012.

[103] Consortium E. P. An integrated encyclopedia of DNA elements in the human genome. Nature 489, 57–74 (2012). 22955616 10.1038/nature11247 PMC3439153

[104] Yu G., Wang L. G. & He Q. Y. ChIPseeker: an R/Bioconductor package for ChIP peak annotation, comparison and visualization. Bioinformatics 31, 2382–2383 (2015). 25765347 10.1093/bioinformatics/btv145

